# Review and Analysis of Peak Tracking Techniques for Fiber Bragg Grating Sensors

**DOI:** 10.3390/s17102368

**Published:** 2017-10-17

**Authors:** Daniele Tosi

**Affiliations:** 1 School of Engineering, Nazarbayev University, Astana 010000, Kazakhstan; daniele.tosi@nu.edu.kz; Tel.: +7-7172-70-5855; 2 Laboratory of Biosensors and Bioinstruments, National Laboratory Astana, Nazarbayev University, Astana 010000, Kazakhstan

**Keywords:** Fiber Bragg Grating (FBG), optical fiber sensors, optical sensors, peak tracking, wavelength shift estimation

## Abstract

Fiber Bragg Grating (FBG) sensors are among the most popular elements for fiber optic sensor networks used for the direct measurement of temperature and strain. Modern FBG interrogation setups measure the FBG spectrum in real-time, and determine the shift of the Bragg wavelength of the FBG in order to estimate the physical parameters. The problem of determining the peak wavelength of the FBG from a spectral measurement limited in resolution and noise, is referred as the peak-tracking problem. In this work, the several peak-tracking approaches are reviewed and classified, outlining their algorithmic implementations: the methods based on direct estimation, interpolation, correlation, resampling, transforms, and optimization are discussed in all their proposed implementations. Then, a simulation based on coupled-mode theory compares the performance of the main peak-tracking methods, in terms of accuracy and signal to noise ratio resilience.

## 1. Introduction

Since the first demonstrations of the photo-induced periodic modulation phenomenon in optical fibers [1,2,3,4], Fiber Bragg Gratings (FBGs) have become established as one of the most prominent technologies used in fiber optic sensors [5]. FBG sensors currently find relevant applications in structural health monitoring [6,7,8,9], civil engineering [8,9,10], oil and gas [11,12,13], aerospace and transportation [14,15], medical devices [16,17,18], nuclear plant monitoring [19,20], high-temperature detection [20,21], and refractive index sensing [22], among other uses.

While the research on FBG sensors until the 2000s has mainly focused on the fabrication of gratings in photosensitive and standard optical fibers [1,2], using techniques such as phase mask [1], interferometric setup [2], direct inscription through pico/femtosecond lasers [23,24], and draw-tower fabrication [25], FBG sensors have seen a progressive standardization of the optical hardware for their interrogation. Thanks to the advances in low-cost spectrometers and tunable lasers, since the last decade two setups have been consolidated for the FBG interrogation: the first setup is based on a broadband source and a spectrometer [1,4,5,26] (often also referred as FBG analyzer), and has been commercialized [27,28,29]; the second setup is based on a fast-scanning tunable laser that sweeps a relatively broad wavelength range (typically 40–100 nm) and a photodetector [30,31,32,33,34,35], and has also been industrialized [36,37].

An FBG behaves as a narrow-band optical reflection filter [1,4,38], which reflects a narrow spectrum around the Bragg wavelength. Thus, it is possible to fabricate several FBGs in the same fiber, each for a different Bragg wavelength, and achieve an array format. FBGs can then discriminate in the optical spectrum domain, in a wavelength-division-multiplexing (WDM) configuration that allows interrogating up to 20–50 sensors in the same fiber [3,4,16,17,18,19,20,21]. In addition, thanks to micro-electromechanical systems (MEMS)-based optical switches [39], it is possible to obtain a multi-channel system, arranged in a time-division-multiplexing (TDM) format in which each FBG array is interrogated by changing the switch position. MEMS switches are available in 1 × 2 and 1 × 4 (the most popular format), up to 1 × 64. The combination of TDM and WDM allows the creation of FBG sensing networks of up to hundreds of sensors.

Interrogators measure the spectrum of the FBG or FBG array in real time. The spectrum of the FBG shifts when a temperature or strain variation is exerted on the grating region, according to a linear relationship [1]. Thus, in order to effectively use an FBG to estimate a temperature or strain variation, it is necessary to estimate the FBG peak wavelength from the FBG spectrum, and in particular the wavelength shift of the FBG from the reference position [1,38]. The peak-tracking routine used to calculate the Bragg wavelength is an important factor in determining the accuracy of the temperature/strain measured with the sensor.

Several peak-tracking techniques have been proposed for the fine estimate of FBG tracking. In the first part of this work, FBG peak tracking methods are reviewed, including direct methods, correlation-based methods, fitting methods, digital oversampling methods, transform-based methods, and optimization-based methods. A thorough review, that extends the peak-tracking analysis released so far [40,41,42,43,44,45,46,47,48,49,50,51], is outlined.

In the second part of the work, the several methods are compared in a simulation-based scenario, in order to analyze the performance. FBGs are simulated with Erdogan’s coupled-mode theory (CMT) [38], and a statistical analysis is used to evaluate the performance of all reviewed methods as a function of the wavelength resolution and signal-to-noise (SNR) ratio of the interrogation setup and on the grating strength coefficient [48]. This analysis allows comparing the different techniques in different working conditions, with the aim of optimizing each method and providing a guideline for their implementation.

The review is arranged as follows: Section 2 describes the theory of and interrogation of FBGs, which constitutes the basis of the peak tracking analysis; Section 3 reviews the peak tracking methods and discusses their implementation into spectral analysis; Section 4 shows a performance analysis for the main peak tracking methods, highlighting the main strengths and criticalities in different working conditions; Section 5 discusses and compares the results of peak tracking methods; finally, Section 6 draws conclusions.

## 2. FBG Sensors and Interrogation

### 2.1. FBG Sensors Theory

The FBG is a periodic modulation of the core refractive index of an optical fiber, which creates a resonant structure that reflects one wavelength, the so-called Bragg wavelength [3,4]. Optically, the FBG behaves as a narrow-band reflective filter, centered at the Bragg wavelength, which reflects a narrow bandwidth while all the other spectral components travel through the FBG without significant losses. Under reference conditions, the Bragg wavelength *λ_B_* can be expressed as [1,38]:

(1) $$\lambda_{B}=2n_{eff}\Lambda$$

where *n_eff_* is the effective refractive index of the optical fiber, and Λ is the period of modulation of the refractive index. Most systems operate around the third optical window with *n_eff_* ≈ 1.5, with FBGs centered around 1520–1600 nm, and therefore Λ typically ranges from 505 nm to 535 nm.

In a uniform FBG, Erdogan’s CMT, which can be directly implemented as in [38] or discretized according to a layer peeling model [52,53], provides a closed-form expression for the grating reflectivity, by solving the core mode coupling between co- and counter-propagating waves. The FBG reflectivity for a uniform grating can be expressed then as:

(2) $$R\left(\lambda \right)=\frac{\sinh^{2}\left(L\sqrt{k^{2}-\sigma^{2}}\right)}{\cosh^{2}\left(L\sqrt{k^{2}-\sigma^{2}}\right)-\frac{\sigma^{2}}{k^{2}}}$$

where: *λ* is the wavelength, *L* is the grating length, and *kL* is a unitless number that typically ranges from 0.1 to 4 and defines the grating strength, both in terms of full-width half-maximum (FWHM) bandwidth, and in terms of maximum reflectivity. The coefficient *σ* has the following expression:

(3) $$\sigma \left(\lambda \right)=\frac{\pi }{\lambda }\delta n_{eff}+2\pi n_{eff}\left(\frac{1}{\lambda }-\frac{1}{\lambda_{B}}\right)$$

where *δn_eff_* is the amplitude of the modulation index modulation.

**Figure 1:**
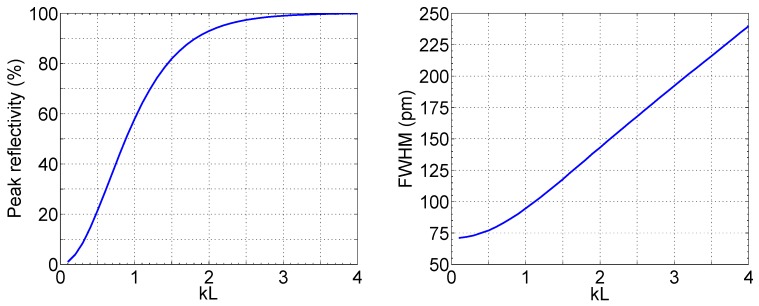
Peak reflectivity (**left**) and FWHM (**right**) for a uniform FBG as a function of the grating strength coefficient *kL*. Parameters: *L* = 1 cm, *n_eff_* = 1.5, *δn_eff_* = 10^−6^, *λ_B_* = 1550 nm.

Figure 1 shows the relationship between the peak reflectivity and the FWHM, as a function of the grating strength coefficient *kL*, for a standard uniform FBG having Bragg wavelength 1550 nm. The peak reflectivity, which defines the amplitude of the spectral slice in correspondence to the Bragg wavelength, grows as tanh^2^ (*kL*) [38], saturating close to 100% for *kL* > 2.5. The FWHM exhibits an almost linear trend for reasonable values of *kL* > 1, up to *kL* = 4.

The FBG can be used as a sensor since both terms *n_eff_* and Λ in Equation (1) are dependent upon strain and temperature [1,4], respectively labeled Δ*ε* and Δ*T*. The result is that the Bragg wavelength *λ_B_* shifts to the value (*λ_B_* + Δ*λ*) in accordance to a linear relationship [4]:

(4) $$\Delta \lambda =k_{\varepsilon }\Delta \varepsilon +k_{T}\Delta T$$

where the coefficients *k_ε_* and *k_T_* incorporate the dependence of both *n_eff_* and Λ. For FBGs operating in the infrared (usually within 1500 nm and 1600 nm, as defined by most commercial devices), typical values of the strain-optic and thermo-optic sensitivities are *k_ε_* ≈ 1 pm/με and *k_T_* ≈ 10 pm/°C [1,2,3,4,5].

An FBG can work as a single-element sensor, providing one measurement output, however, it is possible to maximize the sensing capacity by inscribing an array of FBGs, each having a distinct Bragg wavelength, on the same fiber. Modern inscription methods such as draw-tower fabrication [25,54] can fabricate up to 50 FBGs per single array, with wavelength spacing of ~2 nm between each Bragg wavelength. The resulting spectrum is the combination of all the FBG individual spectra.

In FBG sensing applications, the peak tracking routine estimates the wavelength shift Δ*λ*; then, it is possible to estimate either Δ*ε* or Δ*T* from Equation (4), whereas the sensitivity coefficients *k_ε_* are obtained through a calibration [5].

### 2.2. FBG Interrogation Systems

The current FBG interrogation systems are designed to measure in real time the spectrum of an array of FBGs, with sampling rates ranging from 1–10 Hz for static systems to 1–5 kHz for dynamic systems. The two most popular methods are based on a white-light setup, and a scanning laser system.

**Figure 2:**
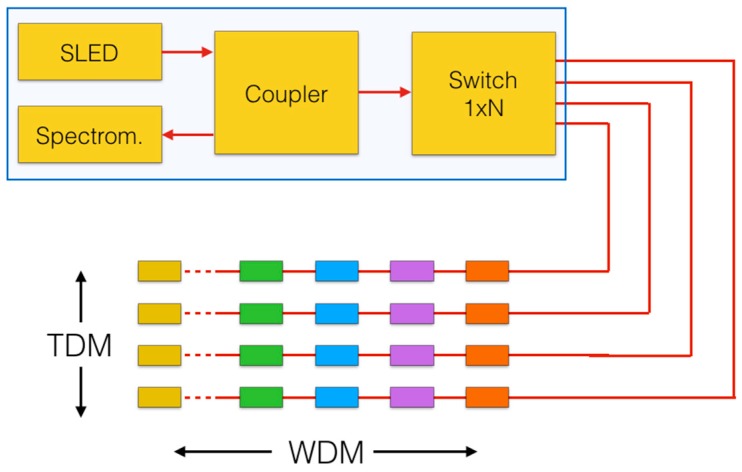
Schematic of white-light interrogation system for FBG sensor networks. Red lines show optical connections; the blue box includes all the building blocks of the interrogator. Colored sensors refer to FBG arrays (one wavelength per color).

The white-light setup is illustrated in Figure 2, as commercialized in [27,28,29]. The light source is a broadband source, typically a superluminescent LED (SLED). Light is routed to the FBG sensing array by means of a coupler, which also allows directing the FBG array backreflection to a charge-coupled device (CCD) spectrometer, that measures the spectral intensity. SLED and spectrometer bandwidths are matched, and usually ranging from 40 nm to 80 nm [29]. A 1 × *N* switch allows multiplexing in the time-domain (TDM) and the combination of TDM and WDM forms a sensing network with a relatively large capacity. The white-light setup has a fast response, limited only by the spectrometer (and, in TDM, by the switching time of the 1 × *N*), but its resolution is limited by the diffraction grating of the spectrometers. Several spectrometers used in FBG sensing have 9-bit (512 pixel) wavelength resolution, almost evenly over a bandwidth of 40 nm or 80 nm [27,29]; thus, the wavelength resolution of the interrogation system is 78 pm or 156 pm, respectively. Therefore, the spectrum of the FBG is coarsely sampled. On the other side, due to the lack of scanning elements and the possibility to set the exposure time of each photodetector element, this interrogation system has a favorable SNR and has a good long-term stability.

**Figure 3:**
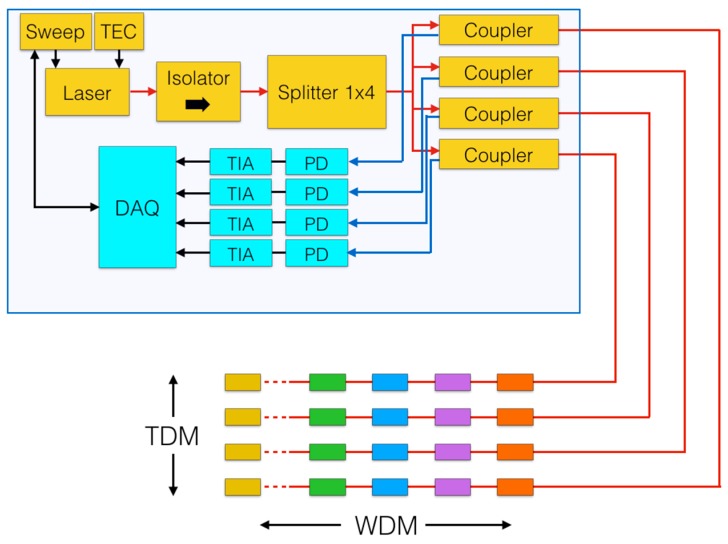
Schematic of scanning-laser interrogation system for FBG sensor networks. Red/blue lines show optical connections, black lines show electrical connections; the blue box includes all the building blocks of the interrogator. Colored sensors refer to FBG arrays (one wavelength per color).

The main alternative to white-light setups is a scanning-laser based system, which is based on a narrow-linewidth laser that rapidly scans a wavelength range. This setup is shown in Figure 3, as industrialized in [36,37]. The light source is a low-power tunable laser, controlled in temperature by a thermo-electric controller (TEC) and in wavelength by a sweep-function generator, which is often implemented in the data acquisition (DAQ) hardware. The laser is followed by an isolator to filter out backreflections. A splitter 1 × *N* (typically 1 × 4 or 1 × 8) is used for multiplexing into multiple channels. Couplers are then used to route light to each FBG array and pack to a photodetector (PD), followed by a transimpedance amplifier (TIA), to obtain the output. The DAQ maintains the synchronization between the sweep-function generator and the array of photodetectors. The main advantage of this setup is the wavelength resolution, as 5–10 pm resolution over up to 100 nm can be obtained [36]. However, the system has an inferior SNR and long-term wavelength stability, due to the use of a laser source.

**Figure 4:**
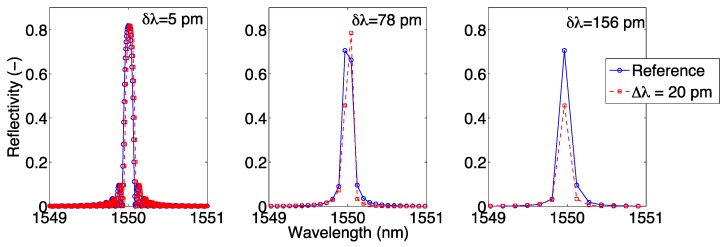
FBG spectra reported in reference condition and for a wavelength shift Δ*λ* = 20 pm, obtained for different values of wavelength resolution *δλ* = 5 pm (**left**), *δλ* = 78 pm (**center**), *δλ* = 156 pm (**right**).

The effect of wavelength resolution is shown in Figure 4, where an FBG spectrum (in ideal noise-free conditions) is simulated using the CMT in Equations (1)–(3) under reference condition and with Δ*λ* = 20 pm wavelength shift, for 3 different values of wavelength grid resolution *δλ*. For a narrow sampling (*δλ* = 5 pm) all the spectral details, including the side lobes [38], are visible in the FBG spectrum; extending the wavelength grid to a coarse resolution (*δλ* = 78–156 pm), the FBG appears as a small set of pixels (where we define pixel as the wavelength spacing between two adjacent wavelengths of the grid), and the spectrum appears to slightly change shape as a result of the under-sampling.

## 3. Review of Peak Tracking Methods

Peak tracking methods for FBGs are reviewed in Table 1. Direct methods perform peak detection on the FBG spectrum by means of direct analysis of the spectrum. Fitting methods perform an interpolation of the FBG spectrum with an analytical function (polynomial, Gaussian, spline), which is then processed to obtain the peak position. Correlation-based methods perform a mutual correlation between the reference FBG spectrum, acquired without any strain or temperature variation, and the measured spectrum. All the previous methods can be applied on the measured FBG spectrum, or (in case of low wavelength resolution) the spectrum can be over-sampled or resampled to artificially expand the resolution *δλ*. Transform-based methods convert, by means of operation such as Fourier Transform, Wavelet, or Karhunen-Loeve Transform, the FBG spectrum in a new domain; the peak search is then implemented in this new domain.

It is important to notice that some methods estimate the Bragg wavelength (*λ_B,e_*) of the measured spectrum, without any a priori knowledge; other methods estimate the wavelength shift (Δ*λ_e_*) from the reference position and require the knowledge of the spectrum in reference condition, which is usually performed at the start of the measurement or during calibration. In practical terms, since Δ*λ* is the only parameter that is needed for the temperature or strain estimation, the two operations have the same effect for most of the applications.

### 3.1. Direct Methods

Direct methods are computationally fast methods that perform simple operations on the reflection spectrum *R* (*λ*) without altering or processing its shape:

#### 3.1.1. Maximum

The maximum method, as described in [41,42], simply estimates the peak wavelength as the wavelength at which the maximum reflectivity is observed using Equation (5) [73]:

(5) $$\lambda_{B,e}={\lambda |}_{R\left(\lambda \right)=R_{\max}}$$

where *R*_max_ is the maximum measured reflectivity. As shown in [42], this method is clearly inefficient in terms of accuracy compared to any other method that has been further implemented.

#### 3.1.2. X-dB Bandwidth (X-BW)

**Figure 5:**
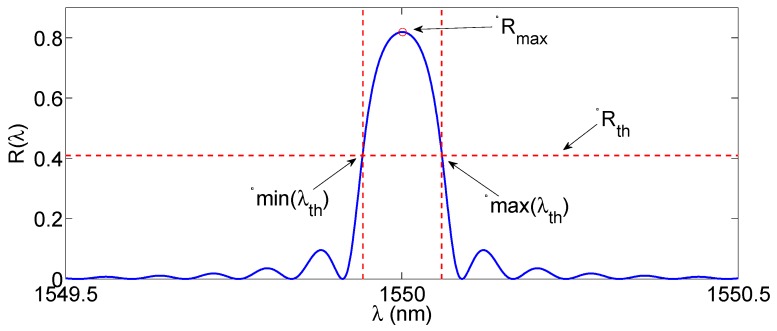
Sketch of the X-dB bandwidth peak-tracking method. The method is depicted for *X* = 3 dB.

The X-dB bandwidth method is one of the most popular techniques for interrogation of FBGs in dense wavelength sampling (*δλ* = 1–5 pm) [42], and is often implemented by the software onboard of commercial interrogators [55]. The approach is sketched in Figure 5 and analytically implemented in Equations (6)–(8), where *X* is in dB [74]:

(6) $$R_{th}=\frac{R_{\max}}{10\log_{10}X}$$

(7) $$\lambda_{th}={\lambda |}_{R\left(\lambda \right)\geq R_{th}}$$

(8) $$\lambda_{B,e}=\min\left(\lambda_{th}\right)+\frac{\max\left(\lambda_{th}\right)-\min\left(\lambda_{th}\right)}{2}$$

The first operation is to set a threshold *R_th_*, having spectral amplitude *X* dB below the maximum measured reflectivity *R*_max_. Then, the set of wavelengths *λ_th_* is calculated as the values for which the reflectivity exceeds *R_th_*; so that they represent the inner bandwidth of the FBG. Finally, the Bragg wavelength is estimated as the center of the inner bandwidth. The value *X* is often set to 3 dB [48]: in this case the X-BW coincides with the evaluation of the center of the FWHM; however it is possible to adjust *X* in order to select a narrower or wider portion of the FBG. In the case of a perfectly symmetrical FBG spectrum, the estimated FBG wavelength coincides with the Bragg wavelength in Equations (1)–(3). In applications where the FBG spectrum is asymmetrical, the estimated wavelength is the center of the X-dB bandwidth and may differ from the Bragg wavelength. However, since the spectra shift without altering their shape, this bias does not alter the definition of Δ*λ*.

#### 3.1.3. Centroid

**Figure 6:**
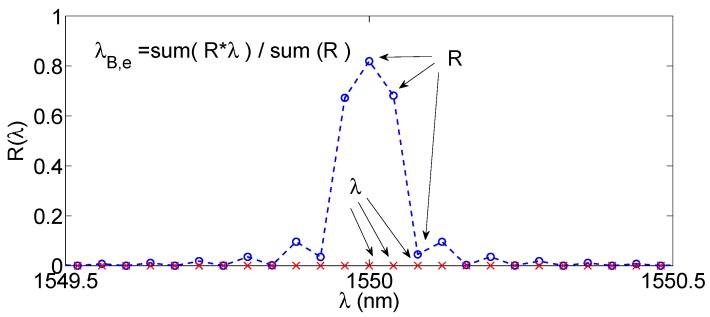
Sketch of the centroid peak-tracking method.

The centroid method, sketched in Figure 6, determines the Bragg wavelength as the center of mass of reflection spectrum of the FBG.

As in [41,42,46,56], the method is algorithmically implemented with a set of two summations:

(9) $$\lambda_{B,e}=\frac{\sum_{n}\lambda_{n}\cdot R\left(\lambda_{n}\right)}{\sum_{n}R\left(\lambda_{n}\right)}$$

where the pixel index n sweeps over the whole set of wavelengths that includes the main portion of the FBG spectrum. Empirically, the Bragg wavelength estimated with the centroid may depend on the width of the spectral range included in the analysis; and this method (like the previous bandwidth tracking) may show a bias of the estimated wavelength versus the Bragg wavelength. However, since we are interested only in the wavelength shift from reference position, this offset does not alter the estimation of Δ*λ*.

### 3.2. Curve Fitting Methods

In a curve-fitting method [75,76,77], the FBG spectrum is interpolated with a function that approximates its shape. Although the CMT theory [38] provides a closed-form expression for the FBG spectrum, it requires a non-linear optimization that is usually not performed (unless in the case of genetic algorithm [52]). Instead, simpler analytical formats that make use of polynomial regression, and depend on a smaller number of parameters are used:

#### 3.2.1. 2nd Order Polynomial

**Figure 7:**
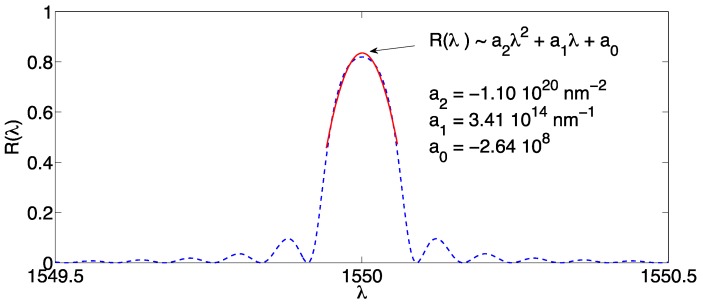
Sketch of the second-order polynomial fit peak tracking; interpolated spectrum is shown in red.

Polynomial fitting methods involve fitting the measured FBG spectrum with a polynomial function, which can be implemented using regression methods [75]. The most classical method is a second order polynomial [42,55,76], thanks to its simple implementation and the unambiguous Bragg wavelength estimation. This method is sketched in Figure 7. In the following formulas:

(10) $$R\left(\lambda \right)\approx a_{2}\lambda^{2}+a_{1}\lambda +a_{0}$$

(11) $$\frac{\partial R}{\partial \lambda }=0\to 2a_{2}\lambda +a_{1}=0$$

(12) $$\lambda_{B,e}=-\frac{a_{1}}{2a_{2}}$$

in first place we approximate the FBG spectrum as a 2nd order polynomial function, determining the set of three coefficients *a*_2_, *a*_1_, *a*_0_. This operation can be performed using a least-squares interpolation method based on quadratic regression. The operation in Equation (10) is performed only on the inner part of the spectrum, which resembles a parabolic function having *a*_2_ < 0; for this reason, this operation is typically preceded by a thresholding as in Equation (6), which is usually performed with *X* = 3 dB [40,55]. Since the spectrum is now approximated as a polynomial of 2nd order and *a*_2_ < 0, there is only one maximum which can be obtained as in Equation (11) by setting the derivative to zero; analytically this is calculated as in Equation (12) without any ambiguity. Software implementation of the 2nd order polynomial fitting method show a good reliability, as the regression does not return badly conditioned polynomials in reasonable SNR regime; this is often implemented in curve-fitting toolboxes [75].

#### 3.2.2. Gaussian Polynomial Fit

**Figure 8:**
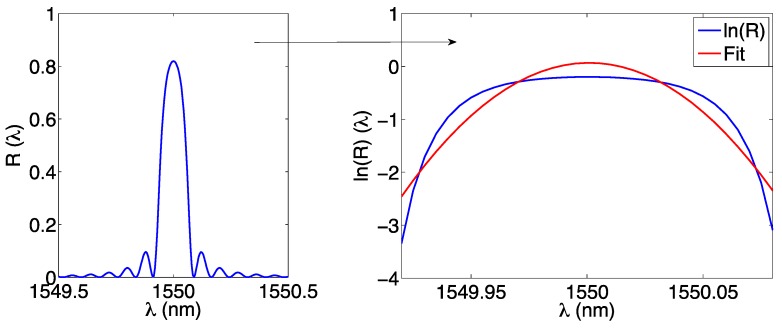
Sketch of the Gaussian→polynomial fitting peak tracking method. (**The left chart**) shows the FBG spectrum, while (**the right chart**) shows the logarithm of the reflection spectrum and its second-order interpolation, zooming into the inner FBG spectral region.

In this method proposed by Chen and Vallan [57], the FBG spectrum is approximated as a Gaussian function; this is shown in Figure 8.

Under this assumption, we can express the FBG spectrum as:

(13) $$R\left(\lambda \right)\approx A\cdot \exp\left[-\frac{{\left(\lambda -\lambda_{0}\right)}^{2}}{2\sigma^{2}}\right]$$

and the Gaussian is determined by the parameters *A*, *σ*, *λ*_0_. Following the steps in [57,78], Equation (13) can be manipulated by dividing the left term by *A*, and taking the logarithm of both terms:

(14) $$\ln\left[R\left(\lambda \right)\right]\approx \ln A-\frac{{\left(\lambda -\lambda_{0}\right)}^{2}}{2\sigma^{2}}$$

which is now a second order polynomial. We can then highlight the 3 coefficient of the polynomial, by making them explicit solving the right term of Equation (14):

(15) $$\begin{array}{l} \ln\left[R\left(\lambda \right)\right]\approx -\frac{1}{2\sigma^{2}}\lambda^{2}+\frac{\lambda_{0}}{\sigma^{2}}\lambda +\left(\ln A-\frac{\lambda_{0}{}^{2}}{2\sigma^{2}}\right)\\ =a_{2}\lambda^{2}+a_{1}\lambda +a_{0} \end{array}$$

and we can highlight that the logarithm of the reflection spectrum can be approximated as a second order polynomial; the coefficients of the polynomial are related to the Gaussian parameters in Equation (13):

(16) $$a_{2}=-\frac{1}{2\sigma^{2}};a_{1}=\frac{\lambda_{0}}{\sigma^{2}};a_{0}=\ln A-\frac{\lambda_{0}{}^{2}}{2\sigma^{2}}$$

Again, we can estimate the Bragg wavelength by imposing that the derivative of ln[*R*(*λ*)] is set to zero:

(17) $$\frac{\partial \left(\ln R\right)}{\partial \lambda }=0\to 2a_{2}\lambda +a_{1}=0$$

and the Bragg wavelength can be estimated as:

(18) $$\lambda_{B,e}=-\frac{a_{1}}{2a_{2}}$$

which is the same result of Equation (12), because still related to a 2nd-order polynomial. Computationally, in this method the second-order regression is applied to the logarithm of the spectrum, implementing directly Equation (15) regardless of the relationship between (*a*_2_, *a*_1_, *a*_0_) and (*A*, *σ*, *λ*_0_), and then the Bragg wavelength is calculated from Equation (18).

#### 3.2.3. Gaussian Fit

The Gaussian fit algorithm, as described in [58], imposes the non-linear regression in Equation (13). Rather than reducing to a polynomial function, the least square method [76] can be applied directly to the spectrum using Equation (13), and the goal is to estimate the parameter *λ*_0_. This method can be implemented using commercially available curve-fitting toolboxes [75].

#### 3.2.4. Higher-Order Polynomial Fitting

The method proposed in [40,46] performs the curve fitting of the whole FBG spectrum with a *N*-th order polynomial function:

(19) $$R\left(\lambda \right)\approx \sum_{n=0}^{N-1}a_{n}\lambda^{n}$$

and estimates the Bragg wavelength by finding the highest maximum of the function used to interpolate *R*(*λ*). This is simply performed by zeroing the derivative:

(20) $$\frac{\partial R}{\partial \lambda }=0\Rightarrow \sum_{n=1}^{N-1}na_{n}\lambda^{n-1}=0$$

In this method, due to the higher order of the polynomial, there are multiple maxima (and minima) in Equation (20); their discrimination is inherently depending on the conditioning of the polynomial in Equation (19).

#### 3.2.5. Spline

The spline method proposed in [40,42,77] allows interpolating the reflection spectrum *R*(*λ*) with a piecewise polynomial function. Typically a cubic spline is used as interpolant, because it ensures a smooth transition between each spectral value up to the second derivative (C^2^ continuity [77,79]). The spline is calculated as follows: given the spectrum *R*(*λ*) sampled on the wavelength grid *λ*_1_, *λ*_2_, …, *λ_N_*, each wavelength acts as the knot of the spline, and one interpolant function *P_i_* is found between each pair of adjacent knots:

(21) $$R\left(\lambda \right)\approx R_{k}\left(\lambda \right)=\underset{\lambda_{k}\leq \lambda \leq \lambda_{k+1}}{\underset{︸}{a_{3,k}\lambda^{3}+a_{2,k}\lambda^{2}+a_{1,k}\lambda +a_{0,k}}},\forall k=1,\ldots ,N-1$$

Since each *k*-th cubic spline function is defined between *λ_k_* and *λ_k_*_+1_, the interpolation is piecewise, and the spectrum can be approximated as:

(22) $$R\left(\lambda \right)\approx \underset{\lambda_{1}\leq \lambda \leq \lambda_{2}}{\underset{︸}{R_{1}\left(\lambda \right)}}\cup \underset{\lambda_{2}\leq \lambda \leq \lambda_{3}}{\underset{︸}{R_{2}\left(\lambda \right)}}\cup \ldots \cup \underset{\lambda_{N-1}\leq \lambda \leq \lambda_{N}}{\underset{︸}{R_{N-1}\left(\lambda \right)}}$$

The spline is thus defined by the 4(*N* − 1) set of *a_i,k_* coefficients in Equation (21). For each spline, four conditions have to be satisfied [77]:

(23) $$\begin{array}{l} R_{k}\left(\lambda_{k}\right)=R\left(\lambda_{k}\right)\\ R_{k}\left(\lambda_{k+1}\right)=R\left(\lambda_{k+1}\right)\\ \frac{\partial R_{k}}{\partial \lambda }\left(\lambda_{k}\right)=\frac{\partial R_{k}}{\partial \lambda }\left(\lambda_{k+1}\right)\\ \frac{\partial^{2}R_{k}}{\partial \lambda^{2}}\left(\lambda_{k}\right)=\frac{\partial^{2}R_{k}}{\partial \lambda^{2}}\left(\lambda_{k+1}\right) \end{array}$$

where the first two equations force the spline interpolant to coincide with the knots, and the last two equations force the two derivatives to be identical in the knots. According to [79,80], Equation (23) can be converted into a matrix and solved.

**Figure 9:**
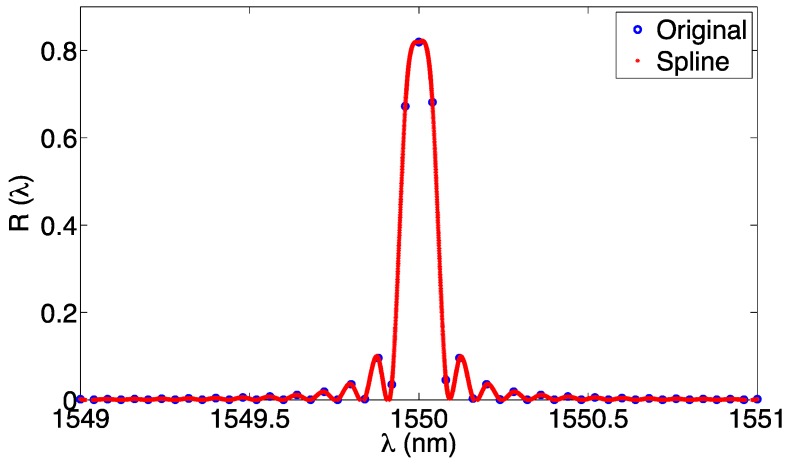
Sketch of the spline peak-tracking method. The original spectrum is sampled on a grid of 40 pm resolution, and interpolated with a cubic spline on a grid with 0.1 pm resolution.

In a spline-based FBG tracking, the FBG spectrum is interpolated with a cubic spline on its grid with resolution *δλ* solving Equation (23) and obtaining the coefficients in Equation (21). Then, by means of Equation (22), it is possible to extend the approximation to all the inter-pixel points, as a matter of fact expanding the spectral estimate to all the values within each knot. The FBG peak is then computed by selecting the maximum value of the spline [40,42]; however, a better alternative is to search for the X-BW bandwidth within the spline-fitted function. The spline process is shown in Figure 9, where a simulated FBG simulated on a 40-pm wavelength grid is interpolated with a spline on a grid of 0.1 pm resolution.

### 3.3. Correlation-Based Methods

Correlation-based methods are based on the calculation of the mutual correlation between the measured spectrum, and the reference spectrum acquired during calibration. These methods do not measure the Bragg wavelength but rather the wavelength shift from the reference to the measured spectrum.

#### 3.3.1. Wavelength-Shifted Mutual Correlation

The method, as described in [48,60], compares the spectrum of the FBG in reference condition *R_ref_*(*λ*) and the measured spectrum *R*(*λ*), which is a replica of *R_ref_*(*λ*) shifted by the quantity Δ*λ*. The method consists of identifying the estimated wavelength shift Δ*λ_e_* as the value of wavelength shift (discretized over the pixels of the spectrometer) for which the mutual correlation is maximum. In terms of implementation, the *m*-th value of mutual correlation is equal to:

(24) $$P_{m}=\sum_{\lambda }R_{ref}\left(\lambda \right)\cdot R\left(\lambda -m\cdot \delta \lambda \right)$$

where *m* is an integer, that allows scanning all the possible wavelength values around the FBG peak. The mutual correlation *P* is a digital function, having maximum value *P*_max_; the estimated wavelength shift Δ*λ_e_* is equal to the value of *mδλ* for which the autocorrelation is maximum:

(25) $$\Delta \lambda_{e}=\delta \lambda \cdot {m|}_{P_{m}=P_{\max}}$$

**Figure 10:**
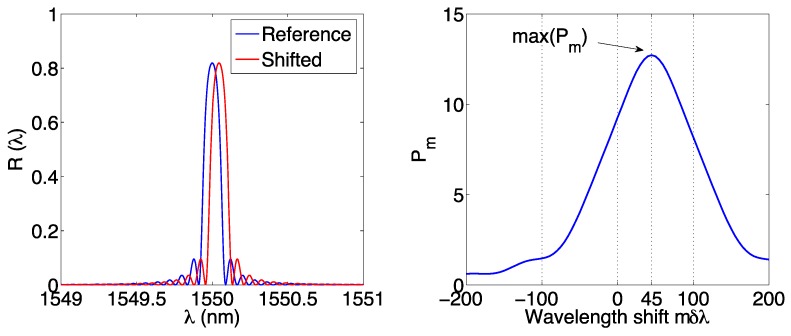
Sketch of the mutual correlation based peak tracking with wavelength shifting. (**Left**) reference and shifted spectra simulated over the wavelength resolution *δλ* = 5 pm; the shifted spectrum has 45 pm wavelength shift. (**Right**) the mutual correlation *P_m_* is calculated over all the wavelength shift values, identifying the wavelength shift as the value that maximizes *P_m_*: this coincides with the wavelength shift.

The process is illustrated in Figure 10. On the left, we consider the reference and measured spectra, the latter being a replica of the original pattern. The autocorrelation *P_m_* has a shape that resembles the inner FBG spectrum, and the peak is located in correspondence of the estimated wavelength shift.

#### 3.3.2. Cross-Correlation

This method is similar to the wavelength-shifted correlation, but does not involve repeating the calculation in Equation (24) for all the values of *m*. The correlation *P*(*λ*) is directly evaluated by computing the cross-correlation of the measured and reference spectra as they are detected [61,62,63]:

(26) $$P\left(\lambda \right)=\sum_{\lambda }R_{ref}\left(\lambda \right)\cdot R\left(\lambda \right)$$

and then the wavelength shift corresponds to the wavelength, on the grid, for which the maximum value of *P* is recorded:

(27) $$\Delta \lambda_{e}=\underset{\Delta \lambda }{\arg\max}P\left(\lambda -\Delta \lambda \right)$$

In a noise-free configuration, solving the cross-correlation with Equations (26) and (27) and the wavelength-shifted correlation in Equations (24) and (25) yield the same results, however a very slight difference may emerge when the spectra are corrupted by noise.

#### 3.3.3. Correlation Polynomial Fit

**Figure 11:**
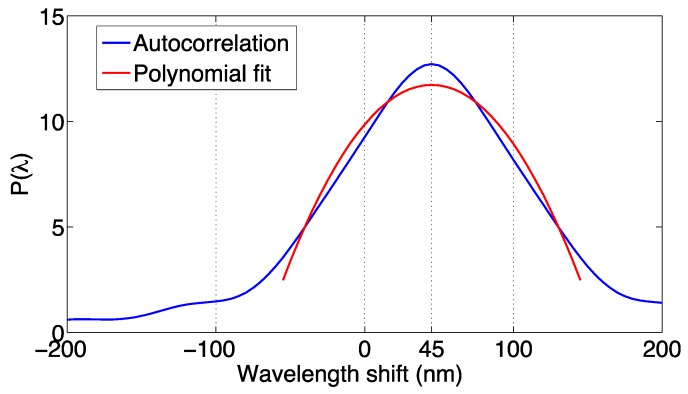
Sketch of the mutual correlation→polynomial fit based peak tracking with wavelength shifting. The autocorrelation is estimated for the same FBG as in Figure 9.

This method, introduced in [63], is based on a sequence of two steps. At first, using Equation (26) the mutual correlation *P*(*λ*) between the reference and measured spectra is computed. Then, the peak-search method (and particularly, the polynomial fitting method in Equations (10)–(12) is used on *P*(*λ*); in practice the same routine for peak search is applied to determine the maximum of the mutual correlation. This method is sketched in Figure 11.

### 3.4. Oversampling

#### 3.4.1. Upsampling

Upsampling is not a technique for peak tracking, but rather complements the other previously introduced methods. The goal of oversampling the FBG spectrum is to artificially expand the resolution of the spectrometer *δλ* into a much denser wavelength grid *δλ_R_*. This operation is critical for methods that are resolution-limited, in which peak resolving is tied to the resolution of the spectrometer. Using oversampling methods [81,82,83,84,85], it is possible to oversample at a very high rate, thus achieving a new wavelength grid *δλ_R_* << *δλ*; the oversampling rate *Q* = *δλ*/*δλ_R_* can be as high as ~150–300, which converts a grid of 78–156 pm [28,29] into a grid with ~1 pm spacing [40,48,55]. However, this grid expansion is artificial, and does not maintain the details of the FBG spectrum. On the other side, it is possible to select a slight grid expansion (*Q* = 4–10) for narrow resolution grid, to slightly improve accuracy.

Upsampling methods artificially expand a digital signal acquired at a lower bit-rate into a higher bit-rate, and is often implemented in audio processing and wireless systems [81,85]. It can also be applied to FBG sensors, as the reflection spectrum of the FBG is regarded as a digital signal of length *L*_1_ and upsampled into a signal with length *L*_2_ = *QL*_1_, multiple of the input signal length. With the upsampling method, the inner spectral samples between one pixel and the next pixel can be determined using a digital filter, obtaining an estimate of the sequence that could have been obtained with a narrower resolution.

The oversampling process is the result of two distinct steps [83]. In the first step (zero-stuffing), the input *R*(*λ*) computed over the wavelengths *λ*_1_, …, *λ_L_*_1_ is rewritten by inserting (*Q* − 1) zero values within each pixel. The new spectral signal *R_S_*(*λ*) thus has the following expression:

(28) $$R_{S}\left[n\right]=\left[\underset{Q\;\mathrm{samples}}{\underset{︸}{R\left(\lambda_{1}\right),0,0,\ldots ,0}},\underset{Q\;\mathrm{samples}}{\underset{︸}{R\left(\lambda_{2}\right),0,0,\ldots ,0}},\ldots ,\underset{Q\;\mathrm{samples}}{\underset{︸}{R\left(\lambda_{L1}\right),0,0,\ldots ,0}}\right]$$

where *n* in this case assumes the meaning of the digital sample. The second step involves a digital low-pass filter, that smoothens the shape of *R_S_*(*λ*) turning it into the best approximation of an oversampled replica of *R*(*λ*). Finite-impulse response (FIR) filters are used for this task [84,85]; the upsampling filter has cut-off digital frequency π/*Q* and gain *Q* (assuming the digital frequency to be in the range within −π and +π). The resample spectrum is then computed as the convolution:

(29) $$R_{R}\left[n\right]=h\left[n\right]*R_{S}\left[n\right]=\sum_{k=1}^{T}h\left[k\right]\cdot R_{S}\left[n-k\right]$$

where *T* is the number of taps in the filter. The implementation of Equation (6) can work for any digital filter; a common approach (implemented in several toolboxes for digital signal processing [83]) is to use a least-square FIR filter.

#### 3.4.2. Resampling

While the classical upsampling implies an integer resampling rate *Q*, resampling allows one to obtain fractional resampling rates *Q* = *Q*_1_/*Q*_2_. The process is similar to oversampling, but two filters and a decimator are employed in cascade [81]. The first filter is a low-pass filter, having gain *Q*_1_ and cut-off digital frequency π/*Q*_1_; the second filter has unitary gain, and cut-off frequency π/*Q*_2_, and is followed by a decimator of size *Q*_2_ that select one sample and discards the subsequent (*Q*_2_ − 1) samples. The most popular implementation of resampling is a polyphase quadrature filter [82], which is included in most popular commercial toolboxes for signal processing, and has been applied to FPGA (field-programmable gate array) for integrated circuit design [86].

**Figure 12:**
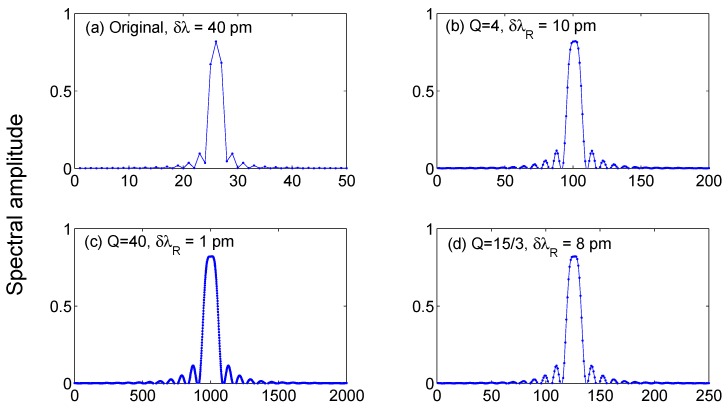
Effect of resampling on a FBG spectrum: the figure shows an FBG spectrum simulated on a wavelength grid of 40 pm resolution filtered through a polyphase resampling filter, showing the digital signals input (**a**) and output (**b**–**d**) of the resampler. (**a**) Original signal; (**b**) polyphase filter with *Q* = 4, bringing the artificial resolution to 10 pm; (**c**) polyphase filter with *Q* = 40, bringing the artificial resolution to 1 pm; (**d**) polyphase filter with *Q* = 15/3, bringing the artificial resolution to 8 pm.

The resampling process for an FBG is outlined in Figure 12, where an FBG spectrum simulated with 40 pm resolution grid is resampled with different rates. The chart shows that several of the spectral features are maintained, as the artificial wavelength grid expands into the new set of samples. Bringing the resolution to 1 pm allows reconstructing several of the side lobes of the FBG, while the spectral profile is smoothed through the low-pass filter. The behavior is similar for an integer or a fractional resampling rate, when a polyphase filter is used. Resampling and upsampling are used in conjunction with the previous methods for peak tracking, applying the method on the resampled spectrum *R_R_* rather than on the original spectrum [55].

### 3.5. Transform-Based Methods

Transforms have been employed for advanced tracking methods, often with the possibility of inter-pixel discrimination, thanks to their capability to move the analysis from the original domain to an alternative domain, where wavelength shifts can be discriminated with a higher sensitivity, without resorting to computationally heavy optimization techniques.

#### 3.5.1. Fast Phase Correlation (FPC)

The method for fast phase correlation has been outlined by Lamberti et al. in [44,64], and requires the measured spectrum *R*(*λ*) as well as the reference spectrum *R_ref_*(*λ*), where *λ*_1_, …, *λ_N_*. At first, the Fourier transforms of both *R*(*λ*) and *R_ref_*(*λ*) are computed through the *N*-size Fast Fourier Transform (FFT):

(30) $$F\left[k\right]=\sum_{n=1}^{N}R\left(\lambda_{n}\right)e^{-j\frac{2\pi nk}{N}}$$

(31) $$F_{ref}\left[k\right]=\sum_{n=0}^{N-1}R_{ref}\left(\lambda_{n}\right)e^{-j\frac{2\pi n\left(k-1\right)}{N}}$$

Then, for each iteration *k* higher than 2, it is possible to set an estimation of the wavelength shift through the phases of *F* and *F_ref_* [64,87]:

(32) $$\Delta \lambda_{e,k-1}=\left(∠F\left[k\right]-∠F_{ref}\left[k\right]\right)\cdot \frac{Nk}{2\pi }\delta \lambda$$

where the operator ∠ is the phase of the complex number, in radians. By scrolling the index *k*, it is possible to obtain subsequent estimates of Δ*λ*: in the implementation of [64], at each iteration *k*, there are (*k* − 1) values of Δ*λ_e_* and the median value is chosen:

(33) $$\Delta \lambda_{e}=\underset{k}{\mathrm{median}}\left(\Delta \lambda_{e,1},\Delta \lambda_{e,2},\ldots ,\Delta \lambda_{e,k-1}\right)$$

The fast phase method presented in [64] allows performing Equation (33) on a number of samples lower than *N*, as the median operator converges with a relatively fast rate.

#### 3.5.2. Linear Phase Operator (LPO)

The linear phase operator method is described in [42] requires a two-step process. A FIR filter *h* is designed having the following *z*-transform:

(34) $$H\left(z\right)=1+z^{-1}-z^{-3}-z^{-4}$$

where the filter coefficients [1, 1, 0, −1, −1] are generated for a 4-th order phase processing filter. The spectrum is then processed through the filter *h*, obtaining the digital output signal *y*[*n*]:

(35) $$y\left[n\right]=h\left[n\right]*R\left[n\right]=\sum_{k=1}^{5}h\left[k\right]\cdot R_{S}\left[n-k\right]$$

**Figure 13:**
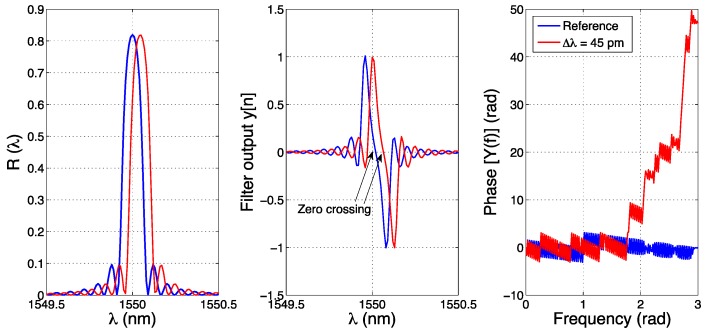
Linear phase operator peak tracking. (**left**) Original spectrum; (**center**) Output of the linear operator filter; (**right**) Phase of the FFT of filter output.

The output of the filter is shown in Figure 13.

It is possible to show that the filter in Equation (34) induces a change of the waveform of the FBG, with two peaks for *y* > 0 and *y* < 0. At this stage, virtually all the previous methods can be used to track the Bragg wavelength. In [42] the authors use a simple method that substantially estimates the Bragg wavelength as the zero-crossing point in proximity of the positive and negative peak of the *y*[*n*] waveform. Extending this method to the algorithm used in particle imaging [88], it is possible to use the FFT of *y*[*n*] to process the shift from the original position; in this case, the phase of the FFT of *y*[*n*] is evaluated, showing a progressive shift of the higher digital frequencies towards larger phase values. This is shown in Figure 13, for the original and wavelength-shifted spectra.

#### 3.5.3. Karhunen-Loeve Transform (KLT)

This method, implemented in [65,66] and resembling principal component analysis [89], is designed for the coarsest wavelength resolution and resolves inter-pixel peak tracking. The KLT is implemented with the following set of calculations [65,66,67]:

(36) $$F\left[k\right]=\sum_{n=1}^{N}R\left(\lambda_{n}\right)e^{-j\frac{2\pi nk}{N}}$$

(37) $$M=\left[\begin{array}{ccccc} F\left[1\right] & F\left[2\right] & F\left[3\right] & \cdots & F\left[N\right]\\ F\left[2\right] & F\left[1\right] & F\left[2\right] & \cdots & F\left[N-1\right]\\ F\left[3\right] & F\left[2\right] & F\left[1\right] & \ddots & \vdots \\ \vdots & \vdots & \ddots & \ddots & F\left[2\right]\\ F\left[N\right] & F\left[N-1\right] & \cdots & F\left[2\right] & F\left[1\right] \end{array}\right]$$

(38) $$M=V\cdot D\cdot V^{-1}$$

(39) $$D=\left[\begin{array}{cccc} \xi_{1} & 0 & \cdots & 0\\ 0 & \xi_{2} & \ddots & \vdots \\ \vdots & \ddots & \ddots & 0\\ 0 & \cdots & 0 & \xi_{N} \end{array}\right]$$

The first step, in Equation (36), calculates the FFT of the reflection spectrum *R*(*λ*) evalauted over *N* wavelengths *λ*_1_, …, *λ_N_*. The FFT output *F*[*k*], *k* = 1, …, *N* is then extended to its Toeplitz matrix ***M*** as in Equation (37) [65]. This procedure allows extending from a one-dimensional to a two-dimensional problem. The KLT is then applied, which consists of the decomposition of the matrix ***M*** into a set of orthonormal basis: the procedure is efficient when it is possible to isolate the contribution of the signal from the noise terms. As shown in Maccone’s works on radar signal processing [90,91], the KLT can be implemented through eigendecomposition as in Equation (38). In this case, the diagonal matrix ***D*** contains, on its main diagonal, the eigenvalues ordered in ascending order such that *ξ*_1_ < *ξ*_2_ < … < *ξ_N_* (and since ***M*** is a Toeplitz matrix, the eigenvalues are all real and positive); ***V*** contains on each column the eigenvector corresponding to each eigenvalue in ***D***. After ***D*** is found in Equation (39), the highest rank eigenvalue *ξ_N_* is extracted [65,90].

**Figure 14:**
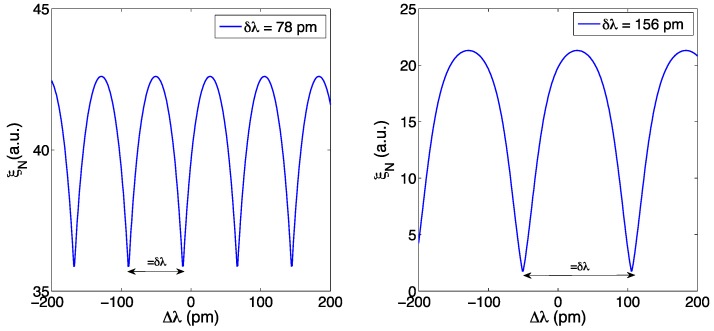
Output of the KLT for a simulated FBG, with wavelength shift Δ*λ* ranging from −200 pm to +200 pm. The charts are obtained for a wavelength grid resolution of 78 pm (**left**) and 156 pm (**right**).

The KLT method is shown in Figure 14 for an FBG and two different sampling resolution (78 pm and 156 pm). The highest rank eigenvalue *ξ_N_* obtained through the process in Equations (36)–(39) is used as a metric for peak tracking. When a wavelength shift Δ*λ* is applied to the FBG, the KLT output exhibits a function that resemble, in waveform, the FBG inner spectrum, and has a periodicity exactly equal to the resolution *δλ*. This accomplishes the goal of expanding the FBG tracking from a resolution-limited grid to an accurate inter-pixel detection; the curve *ξ_N_*(Δ*λ*) can be obtained during calibration [67]. However, the method is limited to a range of Δ*λ* equal to *δλ*/2 without ambiguity, and the function is not linear in trend, thus the method has a variable sensitivity. In order to double the interrogation range to *δλ*, in [66] it is proposed to downsample the FBG spectrum.

The KLT can be combined with the centroid method [65,67], to extend the interrogation to the whole range of Δ*λ*: the centroid method selects the semiperiod of *ξ_N_* (Δ*λ*), providing a first estimate (ballpark); then, the KLT is used for the fine tracking after the semiperiod has been identified.

#### 3.5.4. Capon-KLT

The Capon-KLT method [68] is similar to the KLT, maintaining the calculations in Equations (37)–(39). The FFT in Equation (36) is replaced with a Capon frequency estimation [92,93]: the Capon algorithm allows achieving an improved estimate of the power spectral density of the input signal, and is not limited by the resolution as the FFT [92]. Implementation-wise, the Capon estimator takes the input *R*(*λ*) and estimates *F*[*k*], *k* = 1, …, *T* as:

(40) $$F\left[k\right]=F\left(f_{k}\right)=\frac{1}{a^{H}\left(f_{k}\right)R_{RR}{}^{-1}a\left(f_{k}\right)}$$

where *f_k_* is the *k*-th digital frequency, arbitrarily chosen between 0 and 1. Since the Capon method is based on a FIR filtering [93], the length of *F* and *R* can be different (unlike in the standard KLT). We can set the length of *F*[*k*] to *T*, i.e., *k* = 1, …, *T* corresponding to the range *f*_1_, *f*_2_, …, *f_T_*. Then, the matrix ***R**_RR_* is the *T*-size covariance matrix of the spectrum *R*(*λ*) and a is a *T*-size steering vector with the following definition:

(41) $$a\left(f_{k}\right)=\left[\begin{array}{c} 1\\ e^{-j2\pi f_{k}}\\ e^{-j4\pi f_{k}}\\ \vdots \\ e^{-j2\pi \left(T-1\right)f_{k}} \end{array}\right]$$

The method implemented in [68] for fast computation sets *T* = 5, and *f_k_* = [0.1, 0.125, 0.15, 0.175, 0.2].

#### 3.5.5. Discrete Wavelet Transform (DWT) Denoising

The DWT method, described in [69,70,94], is used to denoise the FBG spectrum, increasing the SNR through one or multiple DWT and inverse (IDWT) filtering. The principle of operation is to use the wavelet functions to filter out part of the noise, while maintaining the shape of the FBG. 1-dimensional Daubechies decimated wavelets db1 and db3 [95] have been used for this task, as they are effective in smoothing out the noise affecting the FBG. As a first step, the FBG spectrum is processed with two filters, determining the approximation coefficients and the details coefficient; in the denoising process, the approximation coefficient of the wavelet is thresholded, removing the coefficients of magnitude higher than the threshold [69]. Then, the reverse process is applied reconstructing the signal. The output of the DWT→thresholding→IDWT can be again filtered one or more times through the same process. In [69], the DWT is also applied to other sensing tasks, such as discriminating spectra of FBG and Fabry-Perot sensors.

**Figure 15:**
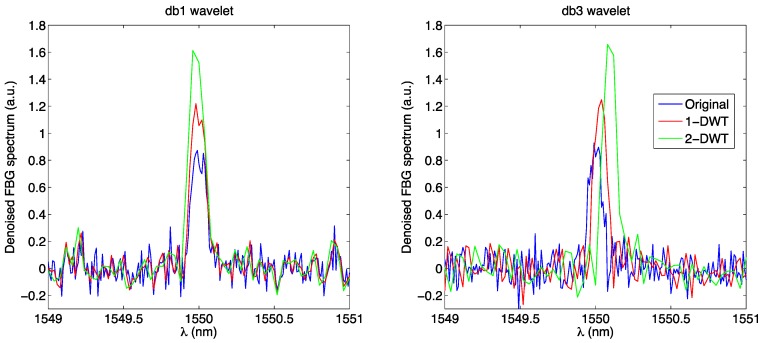
DWT denoising of an FBG spectrum. The original noisy spectrum (blue) is filtered through a DWT→thresholding→IDWT cycle once (red) and twice (green) for denoising. Daubechies wavelets db1 (**left**) and db3 (**right**) have been used.

**Figure 16:**
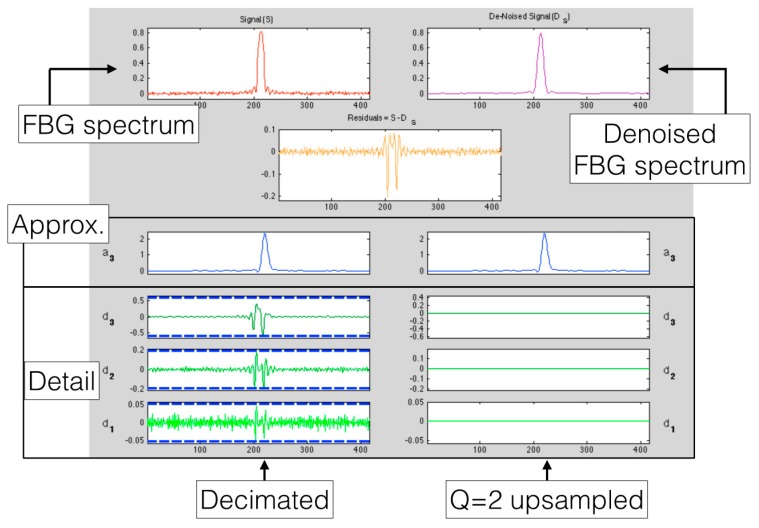
Stationary wavelet analysis of an input spectrum. The upper part shows the noisy FBG spectrum (length *N* = 416), and its denoised estimation through a single db3 operation. The left column shows decimated wavelet waveforms, each having length (*N*/2): the chart shows the approximation *a*_3_, and the detail *d*_1_, *d*_2_, *d*_3_. The right chart shows the upsampled (*Q* = 2) replicas of the decimated coefficients, to restore the original spectral length.

This process is shown in Figure 15, where the FBG spectrum is first filtered through a DWT, then thresholded, and then reversed with the IDWT; this process is incorporated in modern wavelet toolboxes [96], that incorporate the db1 and db3 coefficients. Each filtering through the DWT reduces the samples by half, since the first filter is a decimator: thus, denoising applies well only on the FBG spectra sampled over dense wavelength grid. It is possible to notice that, apart from a delay introduced by the db3 wavelet, multiple DWT filtering reduces the noise on each FBG spectrum, as the FBG peak stands progressively over the noise threshold.The wavelet process of denoising, using a db3 wavelet, is shown in Figure 16 as implemented with the Matlab wavelet analysis [69]. The figure shows the coefficients for approximation (*a*_3_) and the detail coefficients (*d*_1_, *d*_2_, *d*_3_) that are used to progressively denoise the input spectrum. The threshold coefficients have been estimated as 1 for the db1 wavelet, and (1, 416, 0.19) for the db3 wavelet. The left column shows the decimated coefficients, similar to Figure 15, which are the wavelet output; on the right column, a *Q* = 2 upsampler is used to restore the initial length of the signal. The denoised spectrum can be processed with the previously mentioned peak-tracking techniques.

### 3.6. Optimization-Based Techniques

#### 3.6.1. Neural Network

The use of neural network for the peak tracking of FBGs has been introduced in [40,70,71]. The FBG spectrum *R*(*λ*_1_), …, *R*(*λ_N_*) is sampled on an array of *N* wavelengths. Extending the Gaussian-fit concept, the spectrum can be represented as a series of *P* radial-based functions [71]:

(42) $$\varphi \left(\lambda_{p}\right)=e^{-\frac{{\left(\lambda -\lambda_{p}\right)}^{2}}{2\sigma^{2}}}$$

(43) $$R\left(\lambda \right)≃F\left(\lambda \right)=\omega_{1}\varphi \left(\lambda_{1}\right)+\omega_{2}\varphi \left(\lambda_{2}\right)+\ldots +\omega_{P}\varphi \left(\lambda_{P}\right)$$

where Equation (42) shows the Gaussian base function, and Equation (43) shows the function set *F* depending on the weights coefficient string [*ω*_1_, *ω*_2_, …, *ω_P_*]. In [40], an artificial neural network (ANN) is used to estimate the weight coefficients on the base of a cost function that can be represented as follows:

(44) $$J=\sum_{p=1}^{P}{\left[F\left(\lambda_{p}\right)-R\left(\lambda_{p}\right)\right]}^{2}+\gamma \sum_{n=1}^{P}\omega_{n}^{2}$$

The cost function *J* is the sum of the reconstruction error, which is the left term in Equation (44), and the weight-minimization function in the right term, with a coefficient *γ* that balances the two terms. The ANN is applied on Equation (44), until *J* is minimized; then, the largest weight corresponds to the largest radial function, which identifies the estimated Bragg wavelength. The ANN is trained using a neuron by neuron initialization [40], and its implementation has been described in [97].

#### 3.6.2. Genetic Layer Peeling

The layer peeling is technique for FBG analysis, based on a discrete CMT model [49,52,53]. The working principle of this approach is the discretization of a grating into a set of short layers, each defined by its transmission matrix; the multiplication of the cascade of transmission matrices allows determining the whole FBG spectrum. Each transmission matrix contains the reflection and transmission coefficient of each discrete layer, which are derived from the CMT. While the layer peeling is used for the FBG synthesis [52], the reverse layer peeling can be used to estimate the FBG parameters (*λ_B_*, *n_eff_*, *kL*, *δn_eff_*) including the Bragg wavelength. The reverse layer peeling has been implemented with a genetic algorithm, that is able to solve the optimization problem [53]: starting from the FBG spectrum, the algorithm generates a guess for the FBG parameters and continues refining the estimates until the simulated and reconstructed FBG spectra yield the best match. The layer peeling, in this context, can be used for the estimation of *λ_B_* [98].

## 4. Performance Analysis of Main Peak Tracking Methods

### 4.1. Benchmark

A simulative benchmark to insights on the performance analysis of the main peak-tracking method has been designed using the following scenario [40,48], which mimics the process of interrogation that is performed in most FBG applications. The process is substantially a Monte Carlo analysis [99], in which *N_MC_* = 10^6^ FBG spectra with a different wavelength shift are generated with additive noise, and the peak tracking is applied to estimate the wavelength shift from the reference wavelength:

1. The original FBG spectrum is generated on a uniformly spaced wavelength grid with spectral resolution *δλ* using the CMT in Equations (1)–(3). The FBG is generated at the Bragg wavelength *λ_B_* = 1550 nm, and with the following parameters: *L* = 1 cm, *n_eff_* = 1.5, *δn_eff_* = 10^−6^. Whenever not explicit, the grating strength is set as *kL* = 1.5.
2. The original spectrum is corrupted by additive white Gaussian (AWG) noise, setting the SNR level.
3. The peak tracking method is applied for estimating the Bragg wavelength of the FBG in reference condition *λ_B,ref_*.
4. Steps 1–3 are repeated 100 times: the Bragg wavelength *λ_B,ref_* is then selected as the average of the 100 estimates; this process helps reducing the uncertainty on the reference Bragg wavelength.
5. For *n* = 1, 2, …, *N_MC_*:
  - A replica of the original spectrum, having Bragg wavelength by (*λ_B_* + Δ*λ_n_*) is generated on the same wavelength grid. Each Δ*λ_n_* is randomly generated using a uniform probability density function between −20 pm and +80 pm. This corresponds to the measured spectrum.
  - The shifted spectrum is corrupted by AWG noise, with the same SNR as in point 2. This corresponds to the measured spectrum.
  - The peak tracking technique is applied on the measured spectrum, estimating the Bragg wavelength *λ_B,e,n_*.
  - The *n*-th wavelength shift is estimated as: Δ*λ_e_,_n_* = *λ_B,e,n_* − *λ_B,ref_*; this corresponds to the difference between the *n*-th Bragg wavelength estimate and the Bragg wavelength estimate in reference condition.
  - The error in peak tracking is equal to the difference between the estimated and generated wavelength shifts, i.e., *e_n_* = Δ*λ_e_,_n_* − Δ*λ*.
6. The root mean square error (RMSE) of the *N_MC_* estimates is computed, as a metric of the peak-tracking accuracy.

The RMSE is defined as the square root of the *e_n_* terms:

(45) $$RMSE=\sqrt{\frac{\sum_{n=1}^{N_{MC}}e_{n}}{N_{MC}}}$$

The SNR is defined as follows:

(46) $$SNR=\frac{\tanh^{2}\left(kL\right)}{\sigma_{AWG}^{2}}$$

where *σ_AWG_* is the standard deviation of the AWG random process. This definition is more accurate than [48] because it takes into account that the FBG reflectivity has a dependence on *kL*, and it is a good definition for coarse wavelength sampling [29] as the measured peak reflectivity can fluctuate within the pixels.

This simulation takes into account AWG noise. In case of a non-Gaussian noise (as in some spectrometers based on twin 256-pixel hardware [29], which exhibit a higher frequency noise) a whitening filter can be applied [100]. The SNR shows the limit of noise that cannot be further reduced.

### 4.2. X-BW Method

**Figure 17:**
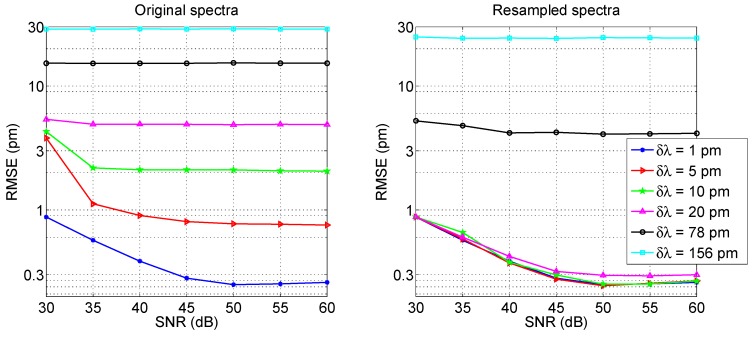
Performance analysis of X-BW peak tracking technique: the RMSE on peak detection is reported as a function of SNR, for different values of sampling grid *δλ* (1 pm, 5 pm, 10 pm, 20 pm, 78 pm, 156 pm). (**Left**) FBG spectra are processed without resampling. (**Right**) FBG spectra have been resampled, on a new grid with *δλ_R_* = 1 pm (corresponding to *Q* = *δλ*/(1 pm)). Threshold: *X* = 3dB.

Figure 17 shows the estimated RMSE for the 3-dB bandwidth tracking method, as a function of both SNR (in a range 30–60 dB) and for different wavelength grids (1, 5, 10, 20, 78 and 156 pm), which represent the resolution of most commercial instruments [27,28,29,36,37].

As a first result, without any resampling, we observe that the resolution is the limiting factor for RMSE, while the SNR does not account for a relevant impact on performance. For a coarse sampling, the SNR has little or no effect on the peak-tracking accuracy: for all SNR values, the RMSE is equal to 28.7 pm for *δλ* = 156 pm, 15.2 pm for *δλ* = 78 pm, and decreases to 4.9 pm for *δλ* = 20 pm, which implies a relatively dense wavelength sampling. Lowering the grid to 10 pm the RMSE ranges from 2.0 pm to 3.8 pm according to SNR. For narrow grids, the sub-pm accuracy is achieved (0.8 to 1.2 pm for *δλ* = 5 pm; 0.3 pm to 0.9 pm for *δλ* = 1 pm). A ~1 pm accuracy can be considered sufficient for most applications, that do not involve precision sensing.

Resampling plays a significant role in improving the accuracy, for all values of wavelength grids. In Figure 17 (right), results are shown for resampled spectra with *δλ_R_* = 1 pm, thus with *Q* ranging from 5 to 156 depending on the spectral density. For *δλ* up to 20 pm, the RMSE improves and overlaps to the values obtained for *δλ* = 1 pm, thus with RMSE ranging from 0.3 pm to 0.9 pm depending on the SNR. For coarse sampling [29], the accuracy improves to 4.2 pm for *δλ* = 78 pm, while for *δλ* = 156 pm, the RMSE is still unacceptable (24.5 pm). From this analysis, it can be concluded that the 3-dB bandwidth tracking is effective in delivering pm-level accuracy for instruments with resolution 20 pm or better, provided that resampling is used. A further resampling to a better grid (*δλ_R_* = 0.1 pm) does not dramatically improve performance and the obtained RMSE gets to ~0.2 pm, while yielding a 10 times larger computational effort.

**Figure 18:**
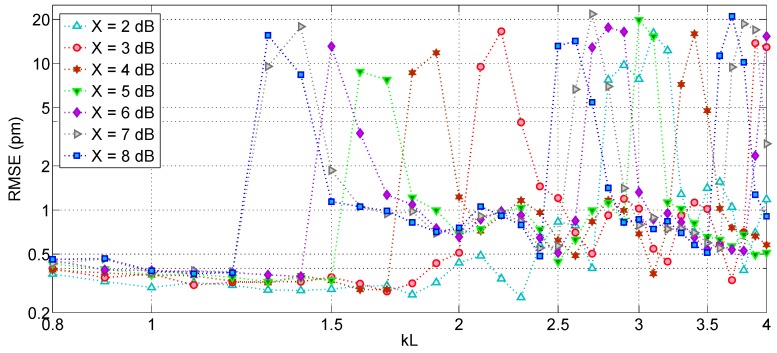
Sensitivity of bandwidth-tracking method to grating strength *kL* and threshold selection *X*. The chart reports RMSE (*kL*) for several values of *X*.

A further analysis can be carried out to evaluate the impact of the FBG bandwidth (*kL* coefficient) and the threshold *X* on the RMSE. Setting a resolution of *δλ* = 20 pm resampled with *Q* = 20, and SNR = 45 dB, the simulation in Figure 18 shows the RMSE as a function of *X* (in range 2–8 dB) and *kL* (in range 0.8–4). The chart shows that regardless of the *X* selection, the RMSE strongly increases by 0.5 to 2 orders of magnitude for larger *kL* values: this is largely due to the effect of side lobes, which are progressively included within the bandwidth and affect the polyphase resampling. The selection of a lower value of *X*, at least for good SNR regime, can improve the performance, as the RMSE recorded for *X* = 2 dB shows the lowest RMSE (0.23 pm) for the widest *kL* range. Values of *X* equal or lower of 1 dB however are ineffective in discriminating the inner FBG spectrum. Overall, the analysis shows that the X-BW peak tracing method is effective with lower *X* values, and with low-reflective FBGs such as [25]. The X-BW peak-tracking method appears to have a performance bound for SNR of approximately 30 dB, as even with resampling lower values of SNR result in excessive RMSE.

### 4.3. Centroid Method

**Figure 19:**
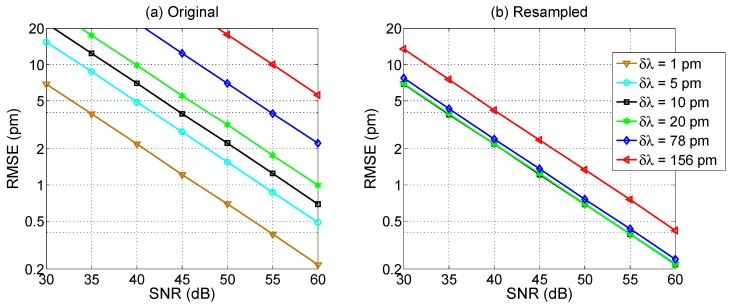
Performance analysis of centroid peak tracking technique: the RMSE on peak detection is reported as a function of SNR, for different values of sampling grid *δλ* (1 pm, 5 pm, 10 pm, 20 pm, 78 pm, 156 pm). (**a**) FBG spectra are processed without resampling. (**b**) FBG spectra have been resampled, on a new grid with *δλ_R_* = 1 pm.

The performance analysis for the centroid method is shown in Figure 19, and exhibits a different trend. Unlike the X-BW method, the centroid accuracy is strongly dependent on both SNR and *δλ* in absence of artificial oversampling. This is shown in Figure 19a, where all the RMSE curves have the same slope: the SNR is inversely proportional to the RMSE, as expected from [46]. For high SNR values (60 dB), the achieved RMSE is close to 0.2 pm for *δλ* = 1 pm, and worsen by a factor 10 as the SNR decreases by 20 dB. The 1-pm RMSE target is achieved for *δλ* up to 20 pm (at SNR = 60 dB), while coarse wavelength grids are not yielding a sufficient RMSE even for the highest SNR.

The situation dramatically changes when resampling is incorporated in the tracking method. As for the previous case, resampling has been performed setting *δλ_R_* = 1 pm. In this case, the wavelength resolution of the spectrometer is not a major limitation, and the RMSE is limited only from the SNR: all values of *δλ* within 1 pm and 20 pm yield the same RMSE, while for *δλ* = 78 pm the results are still very similar, and only for *δλ* = 156 pm an impairment is observed. The 1-pm target is achieved by all methods for SNR higher than 47 dB (52 dB for *δλ* = 156 pm). Overall, the resampling method plays a significant role in improving the results. On the other side, the variation of *kL* coefficient does not significantly alter the RMSE.

### 4.4. Second Order Polynomial Fit

**Figure 20:**
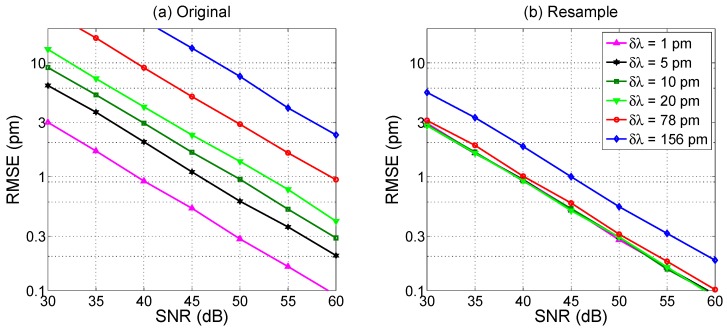
Performance analysis of 2nd order polynomial peak tracking technique: the RMSE on peak detection is reported as a function of SNR, for different values of sampling grid *δλ* (1 pm, 5 pm, 10 pm, 20 pm, 78 pm, 156 pm). (**a**) FBG spectra are processed without resampling. (**b**) FBG spectra have been resampled, on a new grid with *δλ_R_* = 1 pm.

Similarly to the previous analysis, the 2nd order polynomial peak tracking yields a SNR- and grid-dependent RMSE as shown in Figure 20. Again, for non-resampled data the RMSE vs SNR trend exhibits an inversely proportional relationship. More interestingly, resampling also has the effect of saturating the performance to the same level as *δλ* = 1 pm, with the possibility of achieving 0.1 pm RMSE for SNR = 60 dB, 1 pm RMSE for SNR = 35 dB, even for *δλ* = 78 pm. Performance rates show an impairment for *δλ* = 156 pm quantifiable in 5 dB in excess of SNR to achieve the same RMSE.

Overall, the performance of the 2nd order polynomial is better than that of the previous methods, particularly for coarser wavelength resolution. Using resampling, it is possible to obtain pm-level detection with reasonable SNR, approaching 0.2 pm RMSE with outstanding SNR (~55 dB). The *kL* coefficient does not play a significant role in the estimation: while, as shown in Figure 7, the best match between the FBG spectrum and interpolant is achieved for *kL* ~ 1.5, the regression forces the polynomial function to match the FBG center even if the spectrum and interpolant have different shapes.

Compared to the previous centroid method, which is also noise-limited, the slope of the RMSE(SNR) curves is lower for the polynomial fit. This is mainly due to the implementation of the algorithm: while the centroid method is directly affected by the SNR, in this case regression mediates the effect of noise at least for the determination of the polynomial coefficients. Indeed, the centroid method performs better than the polynomial fit for low SNR (<5 dB), in which however the RMSE would be unacceptable for most applications.

### 4.5. Gaussian Fit

**Figure 21:**
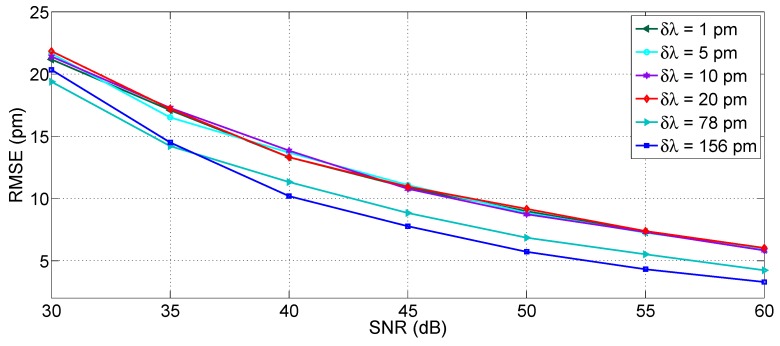
Performance analysis of Gaussian→polynomial peak tracking technique: the RMSE on peak detection is reported as a function of SNR, for different values of sampling grid *δλ* (1, 5, 10, 20, 78, 156 pm); FBG spectra have been resampled, on a new grid with *δλ_R_* = 1 pm.

With the Gaussian polynomial fit method, which requires interpolating the logarithm of the FBG spectrum with a quadratic function, as well as with the Gaussian-fit technique, the obtained RMSE is higher than with polynomial fitting, but does not worsen proportionally to the SNR. The results for the Gaussian polynomial fit method, assuming resampling to *δλ_R_* = 1 pm, are shown in Figure 21; similar results have been obtained for the Gaussian fit. The minimum RMSE for SNR = 60 dB is equal to 2.4 pm, higher than with previous technique, and higher than the typical target for most FBG applications. Counterintuitively, the Gaussian fit method performs better with coarser wavelength grids (156 pm) when it is originally implemented as in [57] and in Figure 21: this is due to the effect of side lobes, which are emphasized by the logarithmic operation and affect the quadratic regression routine, and in undersampled spectra they are slightly flattened out thus reducing disturbance. This effect fades when only the inner FBG portion is used for the regression routine. However, this Gaussian fit method appears not effective in tracking of standard uniform FBGs to the pm-level accuracy, particularly if compared to polynomial fit.

### 4.6. Spline

The spline method has been implemented as in Section 3.2, by applying the spline fit to estimate the set of polynomial coefficients, evaluate the spline on a very narrow grid (0.1 pm as in Figure 9), and use the 3-dB bandwidth to estimate the Bragg wavelength. In this procedure, resampling does not have influence as the spline coefficients (evaluated over the grid *δλ*) are evaluated over a narrower grid.

**Figure 22:**
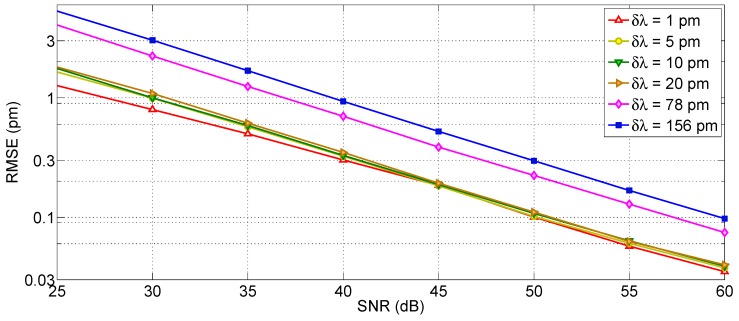
Performance analysis of spline peak tracking technique: the RMSE on peak detection is reported as a function of SNR, for different values of sampling grid *δλ* (1 pm, 5 pm, 10 pm, 20 pm, 78 pm, 156 pm); the spline coefficients have been estimated on the original grid and reevaluated over a grid with 0.1 pm resolution.

The performance analysis is shown in Figure 22. The spline technique returns a RMSE very similar for *δλ* up to 20 pm, and increases for the coarser grids. SNR limits the RMSE in a similar fashion to polynomial fit, as the RMSE(SNR) functions are linear as in Figure 20, but with a slower slope. Quantitatively, the spline method provides the lowest RMSE within the direct and fitting methods, because it comprises the fitting and resampling within its routine. At SNR = 60 dB, the RMSE ranges between 0.03 pm and 0.1 pm; depending on the grid resolution, the RMSE = 1 pm target is achieve for SNR ranging from 27.5 dB and 39 dB. Overall, performances of the spline method are acceptable even for lower values of SNR (25 dB).

### 4.7. Correlation

**Figure 23:**
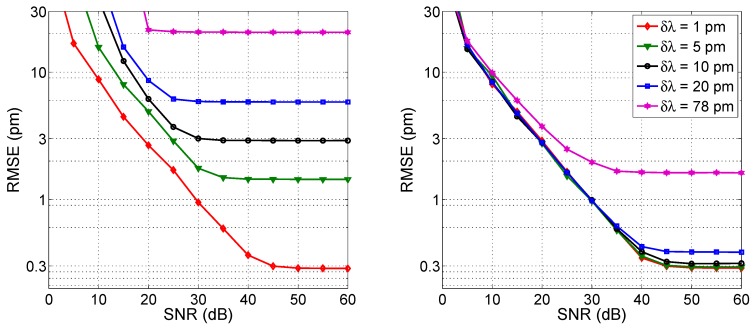
Performance analysis of correlation-based tracking technique: the RMSE on peak detection is reported as a function of SNR, for different values of sampling grid *δλ* (1 pm, 5 pm, 10 pm, 20 pm, 78 pm). (**Left**) FBG spectra are processed without resampling. (**Right**) FBG spectra have been resampled, on a new grid with *δλ_R_* = 1 pm.

The correlation method, as discussed in [60], is designed to operate in low SNR regime, since the correlation process yields an output that is resilient to noise [48,61]. Figure 23 shows the performance analysis, which has been evaluated for SNR decreasing to 0 dB. Without resampling, the method is substantially resolution-limited. For high SNR, the RMSE (SNR) curves are horizontal, while SNR starts to play a substantial role in reducing the accuracy only within 20–30 dB (40 dB for the narrowest resolution). The RMSE in this saturation condition is equal to ~30% of *δλ* (0.3 pm for *δλ* = 1 pm up to 20 pm for *δλ* = 78 pm). However, this regime yields unsatisfactory performance in terms of overall RMSE, as the 1-pm target can be achieved only with 1-pm sampling grid.

Resampling to *δλ_R_* = 1 pm allows improving the performance, as the curves tends to flatten to the *δλ* = 1 pm condition. The target RMSE of 1 pm can be achieved at SNR = 30 dB, while the coarser resolutions returns a higher RMSE. Data for *δλ* = 156 are not shown as they exceed the 30-pm range shown in the chart.

The chart in Figure 23 is derived under the assumption that the reference spectrum is recorded with the same SNR of the measured spectrum. Performance can improve by lowering the SNR (or the resolution) on the reference spectrum as it is acquired: in this case it is possible to maintain horizontal curves for a further ~3 dB of SNR.

**Figure 24:**
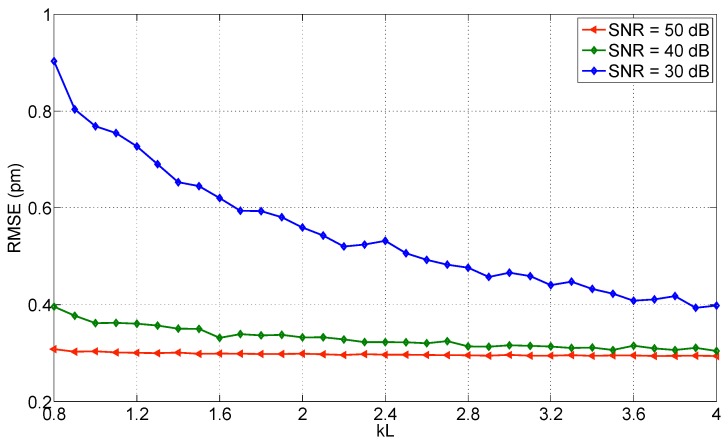
Dependence of the RMSE for the correlation-based tracking method on FBG strength *kL*, for different values of SNR. The FBG spectrum is sampled on a 1-pm grid.

Focusing on the narrowest wavelength grid (1 pm), as the only one that allows achieving a low RMSE, the *kL* coefficient plays a significant role in the performance of the correlation method, as shown in [48], since the correlation is more efficient with wider spectra. Figure 24 shows the variation of the RMSE with different *kL* levels. For the lowest SNR value (30 dB), the RMSE ranges from 0.9 pm (*kL* = 0.8) to 0.4 pm (*kL* = 4); a similar trend is shown for SNR = 40 dB, whereas the RMSE decreases from 0.4 pm to 0.3 pm, while for higher SNR (50 dB) the method is resolution-limited and therefore the RMSE remains almost even over the whole *kL* range.

By applying a polynomial fit to the correlation (correlation→polynomial fit method) performances are slightly better, as a reduction of the RMSE of 1.5 times is observed in the best condition. However, the method does not allow estimating the Bragg wavelength with uncertainty much lower than the wavelength resolution.

### 4.8. Wavelet

**Figure 25:**
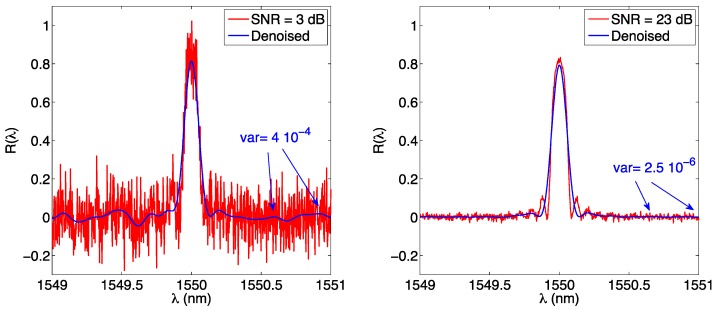
Effect of wavelet denoising on a FBG spectrum sampled over 1 pm wavelength grid. (**left chart**) SNR = 3 dB; (**right chart**) = 23 dB. Denoising has been performed with db3 wavelet.

The wavelet transform has the main benefit of denoising the FBG spectrum when operating in low-SNR regime. The effect of the wavelet is shown in Figure 25, as the db3 wavelet is applied to filter out noise in a 1-pm sampled FBG with SNR equal to 3 dB and 23 dB.

It is not straightforward to evaluate the impact of the wavelet in terms of SNR improvement. In both cases, we observe that the variance outside of the inner FBG region decreases from the initial value to 4.0 × 10^−4^ for SNR = 3 dB and 2.5 × 10^−6^ for SNR = 23 dB, as a result of the low-pass filtering imposed by the wavelet. However, this portion of the spectrum is not used for the analysis. In the inner FBG part, we observe that the wavelet flattens out the side lobes and returns an output that preserves most of the original shape, while removing part of the noise as a result of low-pass processing.

### 4.9. KLT

As in [65,66], the KLT is the method that allows the best inter-pixel discrimination within transform-based techniques. The method, coupled to a centroid tracking for discriminating the semiperiod of operation [65], is intended to application to coarsely sampled spectra: thus, the analysis is focused on *δλ* = 156 pm as in [29]. Unlike the previous method, the KLT does not have a linear sensitivity: thus the obtained RMSE corresponds to the average RMSE obtained over the range of spectral shift Δ*λ*.

**Figure 26:**
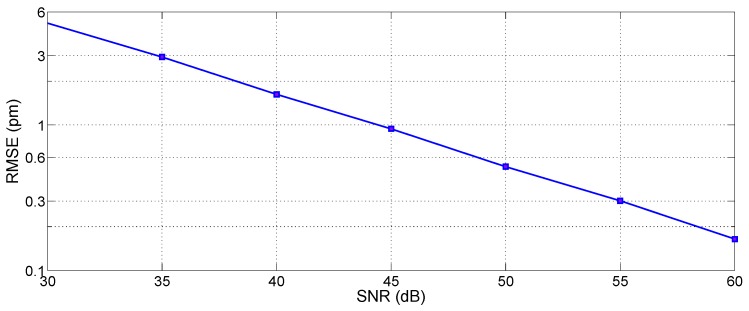
Performance analysis of KLT tracking technique: the RMSE on peak detection is reported as a function of SNR, for coarse grid (*δλ* = 156 pm).

The performance analysis is reported in Figure 26; results are slightly different from [65,66] due to the different SNR definition. The minimum RMSE is 0.18 pm, while the 1-pm target is achieved for a reasonably high SNR (45 dB). Overall, the KLT is able to return an inter-pixel detection even without resampling the signals.

**Figure 27:**
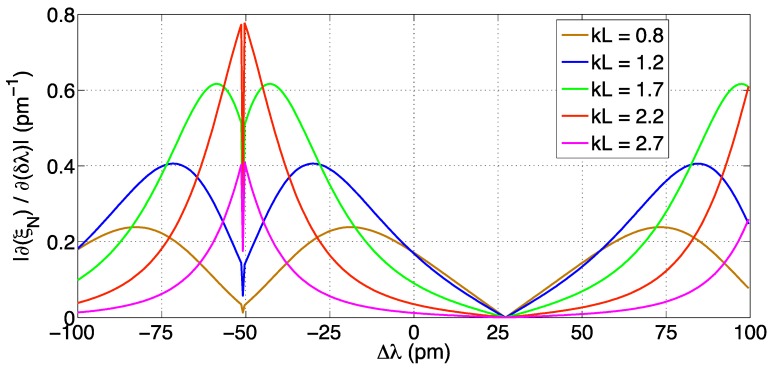
Evaluation of |∂(*ξ_N_*)/∂(Δ*λ*)| as a function of wavelength shift Δ*λ*, for different values of *kL*. Data are obtained for KLT applied to *δλ* = 156 pm.

Since the KLT has a non-linear output, highlighted in Figure 14, the *kL* value as well as the FBG reference wavelength (which can be tuned, for example by pre-straining the grating) can affect the accuracy of the tracking method. To evaluate the sensitivity, Figure 27 shows the derivative of the *ξ_N_* high-rank eigenvalue as a function of the wavelength shift Δ*λ*: the term |∂(*ξ_N_*)/ ∂(Δ*λ*)|is proportional to the RMSE. The result shows the trend similar to [66]. The chart shows a zero in the sensitivity for every value of *kL* at Δ*λ* = 27 pm, and a sensitivity peak with an inner zero of sensitivity for Δ*λ* = −51 pm, which corresponds to the transition between two adjacent semiperiods. Higher sensitivity values are observed for *kL* = 1.7–2.2, while for smaller values the smaller reflectivity imposes a reduction of sensitivity. For high values of *kL* (2.7 or higher) the sensitivity is low as the spectrum is flatter.

## 5. Discussion

In Section 3, the peak tracking methods for FBG detection have been described and classified, while in Section 4 the most significant peak-tracking methods have been analyzed using a simulation-based benchmark to estimate their accuracy as a function of SNR and wavelength resolution grid *δλ*. Quantitatively, the comparison between the different techniques yields some results that can be discussed.

An unambiguous result of the simulation benchmark is that resampling is a key asset in expanding the performance, in the compatible methods. All proposed methods find a great benefit from the upsampling or resampling, and even though the FBG shape can be altered (due to the delay introduced by the upsampler) and the new grid *δλ_R_* is indeed artificial, it is possible to obtain a significant performance boost. While the results obtained without resampling substantially demonstrate that the peak-tracking RMSE is both SNR-limited and resolution-limited for most tracking techniques, when resampling on a much narrower grid it is possible to expand the performance by a great amount and partially compensate the poor resolution of spectrometers. Overall, while some methods are directly dependent on the resolution (for example X-BW and correlation, which are calculated on the wavelength grid and therefore cannot discriminate within two pixels), when resampling is applied the coarse grid appears more as an acceptable penalty on RMSE rather than a limitation. Thus, performance in the following are discussed only in the case of resampled FBG spectra.

Among direct methods, the X-BW method (typically implemented as FWHM) is one the most popular methods on-board of interrogators based on scanning source [36,55]. Its achieved RMSE is ≤ 1 pm for SNR as low as 30 dB, but it is effective only when the resolution is *δλ* ≤ 20 pm. Thus, the method is robust with SNR but cannot be applied to spectrometers and FBG analyzers that have a coarser resolution. The centroid method instead achieves RMSE ≤ 1 pm only for SNR ≥ 45 dB, but it provides better performance for coarse resolution as the 1-pm RMSE level can be achieved also for *δλ* equal 78 pm and 156 pm (for SNR = 47–52 dB), yielding a sensitive penalty only for the coarsest resolution.

The second-order polynomial fit appears as a very efficient method for FBG interrogation. This is due to the fact that, while the spectra (particularly for high *kL* values) do not match a parabolic function, the least square method is effective in minimizing the discrepancy between the Bragg wavelength and the maximum position of the parabolic interpolant, making this method robust. Performance are similar to centroid in terms of SNR resilience, and slightly better in quantitative terms: the RMSE ≤ 1 pm is achieved for SNR ≥ 40 dB, and a penalty is observed for *δλ* = 156 pm. The spline method appears to have excellent performances, as the 1-pm RMSE target is achieved for SNR ≥ 30–37 dB and its performance appear to be more resilient to the width of *δλ*. Overall, the spline has the great benefit of performing interpolation and resampling in a single operation, because once the spline coefficients have been estimated on the original grid, they can be estimated on any arbitrary grid. The Gaussian method provides performance inferior to other fitting methods.

The correlation methods yield inferior performance, and suffer from wavelength grid limitations. Its performances are competitive only on very low SNR regime, similar to X-BW methods.

Among transform-based methods, the KLT obtains the best performance in terms of RMSE. This method, which adapts the principal component analysis to FBG tracking, is effective in converting a small wavelength variation into a significant variation of *ξ_N_*. This method is intended for coarse wavelength sampling, where other methods can fail to deliver a low RMSE. However, its accuracy is dependent on the calibration function, which is not linear; furthermore, a second interrogation system (centroid in [65]) is required to estimate the correct semi-period as the KLT is intrinsically limited to a small spectral range.

The wavelet denoising process, using standard wavelet packets such as Daubechies or Haar that are included in commercial toolboxes [96], is effective in denoising the FBG spectrum. The efficacy is in part due to a low-pass filtering, while the real effect on the FBG spectrum is less evident and in part can change the shape of the spectrum, which may affect the estimate. However, the evidence in [69] suggests that wavelet can be effective in multi-sensor networks, whereas FBGs need to be discriminated from other typologies of sensors.

Qualitatively, the transposition of the simulation-based results in experimental setups depends on too many factors to be properly accounted. At first, the resolution grid for resampling has been chosen as *δλ_R_* = 1 pm, but can be arbitrarily increased or reduced according to the specifications. Secondly, several additional factors affect the grating peak wavelength estimate. These include: distortions and asymmetries of the FBG spectral profile (as opposite to the CMT-simulated spectra [38]); uncertainty on the wavelength grid, due to temperature (in spectrometers [27,29]) or misalignments in the laser source (in tunable scanning lasers [37]); effect of side lobes, which can be apodized; presence of multiple spectral peaks, as sometimes recorded in direct FBG inscription with femtosecond lasers [23]; uncompensated fluctuations of the power through the whole optical fiber path. Furthermore, most FBG sensors operate in WDM-array format, and thus their spectra may be affected by the neighbor FBGs. Qualitatively, these factors affect differently each tracking technique. X-BW, correlation, and fitting methods are generally more robust to amplitude variations, because the calculations are poorly dependent on the spectral amplitude. KLT and transform methods on the other side are less tolerant to non-compensated power fluctuations or spectral deformations of the FBG.

The performance analysis also depend on the target RMSE, which in turn depends on the application for the FBG sensing network. 1-pm accuracy is considered a target in most modern applications, and it corresponds to 1 με for strain sensing or 0.1 °C for temperature detection. For this reason, the resampling rate imposed in the performance analysis has been set to *δλ_R_* = 1 pm. However, some applications require one order of magnitude improvement and in this case it is possible to lower the artificial grid resolution to *δλ_R_* ~ 0.1 pm, which leads to a slight performance improvement, which is still inferior to a factor 10, and often irrelevant when peak tracking is SNR-limited.

The computation time plays a significant role in the application of peak-tracking in real time sensing. For a single FBG, the execution of the peak-tracking routine is performed once for each measurement; in WDM networks with *W* sensors per array, the overall spectra are cut into *W* spectral slices each corresponding to one FBG, and then peak-tracking is applied *W* times, serially (as typically done through DAQ through a computer) or in parallel (as potentially done via FPGA or microcontrollers [86]). Most spectrometers return a 1 kHz sampling rate for the whole spectrum, thus the measurement time is *T_M_* = 1 ms [29]. Calling *T_C_* the computation time for one peak-tracking process, it is important to verify whether *T_M_* >> *WT_C_*: in that case peak tracking does not delay the acquisition speed of the spectrometer. If *T_M_* ~ *WT_C_*, the peak tracking does introduce a delay to peak tracking, without dramatically reducing the speed; if *T_M_* << *WT_C_*, the computational bottleneck is represented by the peak-tracking routine, and therefore the DAQ system is as fast as the FBG peak estimation.

Direct methods are extremely fast: both X-BW and centroid have computation times *T_C_* ≤ 10 μs, well compatible with *W* ~ 100, regardless of the signal length. For quadratic and polynomial fitting, that are based on the same regression routine, the computation time is 1.6 ms for a signal with length 1000, and 0.7 ms for a signal length equal to 50. This result is compatible with dynamic detection (~1 kHz) of a single FBG, or for a static detection (~10 Hz) of an FBG chain. Spline routine has a similar computation time (3.3 ms for length 1000, 1.4 ms for length 50) as the regression procedure is similar, but the spline coefficients are re-evaluated over a narrower grid. The correlation method, implemented as in Equation (26), is nearly as fast as centroid because it is based on a similar computation and thus is well suited for dynamic sensing, while correlation→polynomial fit is limited in speed by the regression. The FPC achieves a computation time in the order of 5 ms. The computation time of KLT is strongly dependent upon signal length: while all the previous methods range from O(*N*) to O(*N* log*N*), the KLT implemented as in [65] is O(*N*^2^ log*N*), and thus its computation time is equal to 1 ms for a signal of length 5, but approaches 10 ms for length 50 and is equal to 0.4 s for *N* = 500. However, since the KLT is mainly intended for coarse sampling, its implementation is mainly addressed to a short-length KLT. Optimization techniques have not been included in performance analysis, because their implementation is extremely slow (several minutes for the genetic algorithms at the base of layer peeling, and tens to hundreds seconds for the neural network): for this reason, these methods appear to be far from being integrated on commercial spectrometer software. Digital resampling, at the base of most techniques, does not significantly impact on computation time, as its computation in Equation (29) is faster than the peak-tracking computations. The calculation of *T_C_* for peak-tracking routines has been performed on a processor 2.6 GHz Intel Core i5 with 16 GB RAM, and peak-tracking methods have been implemented in MATLAB R2013a.

While working with dense sampling does not constitute a major issue in modern systems, thanks to the establishment of tunable scanning lasers and the advent of dense-resolution setups based on semiconductor optical amplifier (SOA) Fabry-Perot methods [101,102], continuous research is performed to reduce the cost of spectrometers and make them more affordable and portable. Methods with inter-pixel capacity, either derived from artificial sampling or inherent in the peak-tracking method (such as the polynomial fitting or the KLT) and with fast computation will support the interrogation of FBGs even in a coarser sampling condition, close to Nyquist digital sampling boundary. In this scenario, it is expected that the combination of a plurality of peak-detection systems can overcome the use of a single technique. For example, the use of wavelet as a denoising method to pre-process the FBG spectrum, followed by a KLT, can improve over current performance limits. In general, transform-based methods represent the state of the art of signal processing, and it is expected that they can find future avenues in FBG peak-tracking, a task somehow similar to particle motion detection or pattern recognition. Computationally, the use of FPGAs can help to parallelize the FBG tracking in sensor arrays.

## 6. Conclusions

In conclusion, the peak-tracking techniques for FBG interrogation have been reviewed and compared. Peak-tracking algorithms are embedded into the software of FBG interrogators, and are an essential part in FBG sensing networks as they allow estimating temperature/strain from FBG spectra. The methods analyzed in this work obtain different performance in terms of RMSE, resilience to SNR, and resilience to wavelength grid resolution.

Overall, the findings of the review and analysis show that direct and fitting methods are well suited for fast FBG interrogation, and the use of resampling makes them effective even with coarse wavelength grids. Correlation-based methods instead are extremely resilient to SNR. Among transform-based methods, the KLT provides a good potential for precision-sensing. All these methods have fast implementation, and can be operated for real-time sensing without reducing the sampling rate of the interrogation hardware.

## Figures and Tables

**Table 1:** List of peak-tracking techniques reviewed in Section 3.

Type	Method	Main References
Direct	Maximum	[41,42]
	X-dB bandwidth	[42,47,55]
	Centroid	[41,42,46,56]
Curve fitting	2nd order polynomial	[42,46]
	Gaussian→polynomial	[57]
	Gaussian fit	[58]
	High-order polynomial	[40,42]
	Spline	[42,59]
Correlation	Wavelength shifted correl.	[48,60]
	Cross-correlation	[61,62]
	Correlation→polynomial	[63]
Oversampling ^1^	Upsampling	[48,55]
	Resampling	[48,55]
Transform	Fast phase correlation	[44,64]
	Linear-phase operator	[42]
	Karhunen-Loeve Transform	[65,66,67]
	Capon-KLT	[68]
	Wavelet	[69,70]
Optimization	Neural network	[40,71,72]
	Genetic layer peeling	[49,53]

^1^ Overampling routines are not used directly for FBG tracking, but in connection with the previous peak-tracking methods.

## References

[1] Othonos A., Kalli K. (1999). Fiber Bragg Gratings: Fundamentals and Applications.

[2] Meltz G., Morey W.W., Glenn W.H. (1989). Formation of Bragg gratings in optical fibers by a transverse holographic method. Opt. Lett..

[3] Rao Y.J. (1997). In-fibre Bragg grating sensors. Meas. Sci. Technol..

[4] Kersey A.D., Davis M.A., Patrick H.J., LeBlanc M., Koo K.P., Askins C.G., Putnam M.A., Friebele E.J. (1997). Fiber grating sensors. J. Lightwave Technol..

[5] Udd E., Spillman W.B. (2011). Fiber Optic Sensors: An Introduction for Engineers and Scientists.

[6] Kinet D., Megret P., Goossen K.W., Qiu L., Heider D., Caucheteur C. (2014). Fiber Bragg grating sensors toward structural health monitoring in composite materials: Challenges and solutions. Sensors.

[7] Chan T.H., Yu L., Tam H.Y., Ni Y.Q., Liu S.Y., Chung W.H., Cheng L.K. (2006). Fiber Bragg grating sensors for structural health monitoring of Tsing Ma bridge: Background and experimental observation. Eng. Struct..

[8] Lopez-Higuera J.M., Rodriguez Cobo L., Quintela Incera A., Cobo A. (2011). Fiber optic sensors in structural health monitoring. J. Lightwave Technol..

[9] Leng J., Asundi A. (2003). Structural health monitoring of smart composite materials by using EFPI and FBG sensors. Sens. Actuators A Phys..

[10] Rezayat A., De Pauw B., Lamberti A., El-Kafafy M., Nassiri V., Ertveldt J., Arroud G., Vanlanduit S., Guillaume P. (2016). Reconstruction of impacts on a composite plate using fiber Bragg gratings (FBG) and inverse methods. Compos. Struct..

[11] Brown G.A., Hartog A. (2002). Optical fiber sensors in upstream oil & gas. J. Pet. Technol..

[12] Zhao Y., Liao Y., Lai S. (2002). Simultaneous measurement of down-hole high pressure and temperature with a bulk-modulus and FBG sensor. IEEE Photonics Technol. Lett..

[13] Wang Q., Zhang L., Sun C., Yu Q. (2008). Multiplexed fiber-optic pressure and temperature sensor system for down-hole measurement. IEEE Sens. J..

[14] Lamberti A., Chiesura G., Luyckx G., Degrieck J., Kaufmann M., Vanlanduit S. (2015). Dynamic strain measurements on automotive and aeronautic composite components by means of embedded fiber Bragg grating sensors. Sensors.

[15] Schroeder K., Ecke W., Apitz J., Lembke E., Lenschow G. (2006). A fibre Bragg grating sensor system monitors operational load in a wind turbine rotor blade. Meas. Sci. Technol..

[16] Dziuda L., Skibniewski F.W., Krej M., Lewandowski J. (2012). Monitoring respiration and cardiac activity using fiber Bragg grating-based sensors. IEEE Trans. Biomed. Eng..

[17] Arkwright J.W., Blenman N.G., Underhill I.D., Maunder S.A., Spencer N.J., Costa M., Brookes S.J., Szczesniak M.M., Dinning P.G. (2012). Measurement of muscular activity associated with peristalsis in the human gut using fiber Bragg grating arrays. IEEE Sens. J..

[18] Tosi D., Macchi E.G., Braschi G., Gallati M., Cigada A., Poeggel S., Leen G., Lewis E. (2014). Monitoring of radiofrequency thermal ablation in liver tissue through fibre Bragg grating sensors array. Electron. Lett..

[19] Fernandez A.F., Gusarov A.I., Bodart S., Lammens K., Berghmans F., Decre M., Me P., Blondel M., Delchambre A. (2002). Temperature monitoring of nuclear reactor cores with multiplexed fiber Bragg grating sensors. Opt. Eng..

[20] Mihailov S.J. (2012). Fiber Bragg grating sensors for harsh environments. Sensors.

[21] Zhang B., Kahrizi M. (2007). High-temperature resistance fiber Bragg grating temperature sensor fabrication. IEEE Sens. J..

[22] Gu B., Qi W., Zheng J., Zhou Y., Shum P.P., Luan F. (2014). Simple and compact reflective refractometer based on tilted fiber Bragg grating inscribed in thin-core fiber. Opt. Lett..

[23] Chah K., Kinet D., Wuilpart M., Megret P., Caucheteur C. (2013). Femtosecond-laser-induced highly birefringent Bragg gratings in standard optical fiber. Opt. Lett..

[24] Poeggel S., Duraibabu D., Tosi D., Leen G., Lewis E., Lacraz A., Hambalis M., Koutsides C., Kalli K. Novel FBG femtosecond laser inscription method for improved FPI sensors for medical applications. Proceedings of the IEEE Sensors Conference.

[25] Lindner E., Hartung A., Hoh D., Chojetzki C., Schuster K., Bierlich J., Rothhardt M. Trends and future of fiber Bragg grating sensing technologies: Tailored draw tower gratings (DTGs). Proceedings of the SPIE Photonics Europe.

[26] Jiang Y., Ding W. (2011). Recent developments in fiber optic spectral white-light interferometry. Photonic Sens..

[27] Bayspec FBGA. http://www.bayspec.com/telecom-fiber-sensing/fbga-systems/.

[28] FBGS International FBGG-Scan Interrogators. http://www.fbgs.com/products/measurement-devices/fbg-scan-704d/804d/.

[29] Ibsen Photonics Interrogation Monitors. http://www.ibsenphotonics.com.

[30] Kwon Y.S., Ko M.O., Jung M.S., Park I.G., Kim N., Han S.P., Ryu H.C., Park K.H., Jeon M.Y. (2013). Dynamic sensor interrogation using wavelength-swept laser with a polygon-scanner-based wavelength filter. Sensors.

[31] Yun S.H., Richardson D.J., Kim B.Y. (1998). Interrogation of fiber grating sensor arrays with a wavelength-swept fiber laser. Opt. Lett..

[32] Mohanty L., Koh L.M., Tjin S.C. (2006). Fiber Bragg grating microphone system. Appl. Phys. Lett..

[33] Bezombes F.A., Lalor M.J., Burton D.R. (2007). Contact microphone using optical fibre Bragg grating technology. J. Phys. Conf. Ser..

[34] Wilson A., James S.W., Tatam R.P. (2001). Time-division-multiplexed interrogation of fibre Bragg grating sensors using laser diodes. Meas. Sci. Technol..

[35] Tosi D., Olivero M., Perrone G. (2008). Low-cost fiber Bragg grating vibroacoustic sensor for voice and heartbeat detection. Appl. Opt..

[36] Micron Optics Interrogators. http://www.micronoptics.com.

[37] HBM Fibersensing Braggmeter. https://www.hbm.com/en/2322/optical-interrogators-from-hbm-fibersensing/.

[38] Erdogan T. (1997). Fiber grating spectra. J. Lightwave Technol..

[39] Sercalo MEMS Switches. http://www.sercalo.com.

[40] Negri L., Nied A., Kalinowski H., Paterno A. (2011). Benchmark for peak detection algorithms in fiber Bragg grating interrogation and a new neural network for its performance improvement. Sensors.

[41] Dyer S.D., Williams P.A., Espejo R.J., Kofler J.D., Etzel S.M. (2005). Fundamental limits in fiber Bragg grating peak wavelength measurements. Proc. SPIE.

[42] Bodendorfer T., Muller M., Hirth F., Koch A. Comparison of different peak detection algorithms with regards to spectrometric fiber Bragg grating interrogation systems. Proceedings of International Symposium on Optomechatronic Technologies (ISOT).

[43] Lamberti A., Vanlanduit S., De Pauw B., Berghmans F. (2014). Influence of fiber Bragg grating spectrum degradation on the performance of sensor interrogation algorithms. Sensors.

[44] Lamberti A., Vanlanduit S., De Pauw B., Berghmans F. (2014). Peak detection in fiber Bragg grating using a fast phase correlation algorithm. Proc. SPIE.

[45] Kersey A.D., Morey W.W., Berkoff T.A. (1993). Fiber-optic Bragg grating strain sensor with drift-compensated high-resolution interferometric wavelength-shift detection. Opt. Lett..

[46] Ezbiri A., Kanellopoulos S.E., Handerek V.A. (1998). High resolution instrumentation system for fiber-Bragg grating aerospace sensors. Opt. Commun..

[47] Posseti G.R.C., Kamikawachi R.C., Muller M., Fabris J.L. (2002). Metrological evaluation of optical fiber grating-based sensors: An approach toward the standardization. J. Lightwave Technol..

[48] Tosi D., Olivero M., Perrone G. (2012). Performance analysis of peak tracking techniques for fiber Bragg grating interrogation systems. J. Microw. Optoelectron. Electromagn. Appl..

[49] Gill A., Peters K., Studer M. (2004). Genetic algorithm for the reconstruction of Bragg grating sensor strain profiles. Meas. Sci. Technol..

[50] Jiang J., Liu T., Liu K., Zhang Y. (2012). Investigation of peak wavelength detection of fiber Bragg grating with sparse spectral data. Opt. Eng..

[51] Harasim D., Gulbahar Y. Improvement of FBG peak wavelength demodulation using digital signal processing algorithms. Proceedings of the XXXVI Symposium on Photonics Applications in Astronomy, Communications, Industry, and High-Energy Physics Experiments.

[52] Skaar J., Wang L., Erdogan T. (2001). On the synthesis of fiber Bragg gratings by layer peeling. J. Lightwave Technol..

[53] Skaar J., Risvik K.M. (1998). A genetic algorithm for the inverse problem in synthesis of fiber gratings. J. Lightwave Technol..

[54] Johnson D. (2012). Novel Optical Fibers-Draw-tower process creates high-quality FBG arrays. Laser Focus World.

[55] Micron Optics Enlight Software. http://www.micronoptics.com/products/sensing-solutions/software/.

[56] Atkins C.G., Putnam M.A., Friebele E.J. (1995). Instrumentation for interrogating many-element fiber Bragg grating arrays. Proc. SPIE.

[57] Chen W., Vallan A. Applications of a Fast FBG Interrogation System for Real-Time Thermal and Structural Monitoring. Proceedings of the IEEE 1st International Forum on Research and Technologies for Society and Industry: Leveraging a Better Tomorrow (RTSI).

[58] Lee H.W., Park H.J., Lee J.H., Song M. (2007). Accuracy improvement in peak positioning of spectrally distorted fiber Bragg grating sensors by Gaussian curve fitting. Appl. Opt..

[59] De Sousa M.J., Costa J.C., De Souza R.M., Pantoja R.V. (2011). FBG optimization using spline encoded evolution strategy. J. Microw. Optoelectron. Electromagn. Appl..

[60] Caucheteur C., Chah K., Lhomme F., Blondel M., Megret P. (2004). Autocorrelation demodulation technique for fiber Bragg grating sensor. IEEE Photonics Technol. Lett..

[61] Gong J.M., MacAlpine J.M., Chan C.C., Jin W., Zhang M., Liao Y.B. (2002). A novel wavelength detection technique for fiber Bragg grating sensors. IEEE Photonics Technol. Lett..

[62] Gong J.M., Chan C.C., Jin W., MacAlpine J.M.K., Zhang M., Liao Y.B. (2002). Enhancement of wavelength detection accuracy in fiber Bragg grating sensors by using a spectrum correlation technique. Opt. Commun..

[63] Huang C., Jing W., Liu K., Zhang Y., Peng G. (2007). Demodulation of fiber Bragg grating sensor using cross-correlation algorithm. IEEE Photonics Technol. Lett..

[64] Lamberti A., Vanlanduit S., de Pauw B., Berghmans F. (2014). A novel fast phase correlation algorithm for peak wavelength detection of fiber Bragg grating sensors. Opt. Express.

[65] Tosi D. (2015). KLT-based algorithm for sub-pm accurate FBG tracking with coarse wavelength sampling. Photonics Technol. Lett..

[66] Tosi D. (2015). Advanced interrogation of fiber-optic Bragg grating and Fabry-Perot sensors with KLT analysis. Sensors.

[67] Hervás J., Tosi D., García-Miquel H., Barrera D., Fernández-Pousa C.R., Sales S. (2017). KLT-Based Interrogation technique for FBG multiplexed sensor tracking. J. Lightwave Technol..

[68] Tosi D. (2017). Improved KLT Algorithm for high-precision wavelength tracking of optical fiber bragg grating sensors. J. Sens..

[69] Wong A.C., Peng G.D. Applications of Discrete Wavelet Transform in Optical Fibre Sensing. https://www.intechopen.com/books/discrete-wavelet-transforms-biomedical-applications/applications-of-discrete-wavelet-transform-in-optical-fibre-sensing.

[70] Zhaoxia W., Haili Y. Fiber Bragg Grating Peak Wavelength Detection Technique Based on Wavelet Analysis. Proceedings of the IEEE International Conference on Internet Computing & Information Services (ICICIS).

[71] Paterno A.S., Silva J.C.C., Milczewski M.S., Arruda L.V.R., Kalinowski H.J. (2006). Radial-basis function network for the approximation of FBG sensor spectra with distorted peaks. Meas. Sci. Technol..

[72] Chan C.C., Shi C.Z., Jin W., Wang D.N. (2003). Improving the wavelength detection accuracy of FBG sensors using an ADALINE network. IEEE Photonic Technol. Lett..

[73] Geiman B., Bohs L., Anderson M., Breit S., Trahey G. A Comparison of Algorithms for Tracking Sub-pixel Speckle Motion. Proceedings of the IEEE Ultrasonics Symposium.

[74] Liu Q., Qiao X., Jia Z.A., Fu H. (2016). Spectra power and bandwidth of fiber Bragg grating under influence of gradient strain. Photonic Sens..

[75] Matlab, Mathworks Curve Fitting Toolbox. https://www.mathworks.com/products/curvefitting.html.

[76] Lancaster P., Salkauskas K. (1986). Curve and Surface Fitting: An Introduction.

[77] Dierckx P. (1995). Curve and Surface Fitting with Splines.

[78] Guo H. (2011). A simple algorithm for fitting a gaussian function [DSP tips and tricks]. IEEE Signal Proc. Mag..

[79] Cuche E., Marquet P., Depeursinge C. (2000). Aperture apodization using cubic spline interpolation: Application in digital holographic microscopy. Opt. Commun..

[80] Bartels R.H., Beatty J.C., Barsky B.A. (1987). An Introduction to Splines for Use in Computer Graphics and Geometric Modeling.

[81] Yap B.W., Rani K.A., Rahman H.A., Fong S., Khairudin Z., Abdullah N.N. An Application of Oversampling, Undersampling, Bagging and Boosting in Handling Imbalanced Datasets. Proceedings of the First International Conference on Advanced Data and Information Engineering (DaEng-2013).

[82] Rothweiler J. Polyphase quadrature filters—A new subband coding technique. Proceedings of the IEEE International Conference on Acoustics, Speech, and Signal Processing (ICASSP).

[83] Clark C.L. (2005). LabVIEW Digital Signal Processing.

[84] Harris F.J., Dick C., Rice M. (2003). Digital receivers and transmitters using polyphase filter banks for wireless communications. IEEE Trans. Microw. Theory Tech..

[85] Vetterli M. (1984). Multi-dimensional sub-band coding: Some theory and algorithms. Signal Process..

[86] Wang Y., Negri L.H., Cervi G., De Oliveira V., Kalinowski H.J., Paterno A.S. Multiplexed FBG Optical Instrumentation Using an FPGA-Based System. Proceedings of the Latin America Optics and Photonics Conference.

[87] Eckstein A.C., Charonko J. (2008). Phase correlation processing for DPIV measurements. Exp. Fluids.

[88] Beraldin J.A., Cournoyer L., Rioux M., Blais F., El-Hakim S.F., Godin G. Object model creation from multiple range images: Acquisition, calibration, model building and verification. Proceedings of the International Conference on Recent Advances in 3-D Digital Imaging and Modeling.

[89] Stewart S., Ivy M.A., Anslyn E.V. (2014). The use of principal component analysis and discriminant analysis in differential sensing routines. Chem. Soc. Rev..

[90] Maccone C. (2005). Advantages of Karhunen-Loeve transform over fast Fourier transform for planetary radar and space debris detection. Acta Astronaut..

[91] Maccone C. (1994). Telecommunications, KLT and Relativity.

[92] Zaknich A. (2006). Principles of Adaptive Filters and Self-Learning Systems.

[93] Capon J. (1969). High-resolution frequency-wavenumber spectrum analysis. Proc. IEEE.

[94] Gangopadhyay T.K., Chakravorti S., Bhattacharya K., Chatterjee S. (2005). Wavelet analysis of optical signal extracted from a non-contact fibre-optic vibration sensor using an extrinsic Fabry-Perot interferometer. Meas. Sci. Technol..

[95] Daubechies I. (1992). Ten Lectures on Wavelets.

[96] Matlab, Mathworks Wavelet Toolbox. https://www.mathworks.com/products/wavelet.html.

[97] Omondi A.R., Rajapakse J.C. (2006). FPGA Implementations of Neural Networks.

[98] Shi C.Z., Chan C.C., Jin W., Liao Y.B., Zhou Y., Demokan M.S. (2003). Improving the performance of a FBG sensor network using a genetic algorithm. Sens. Actuators A Phys..

[99] Robert C.P. (2004). Monte Carlo Methods.

[100] Benedetto S., Biglieri E. (1999). Principles of Digital Transmis-Sion with Wireless Applications.

[101] Chung W.H., Tam H.-Y., Wai P.K.A., Khandelwal A. (2005). Time-and wavelength-division multiplexing of FBG sensors using a semiconductor optical amplifier in ring cavity configuration. IEEE Photonics Technol. Lett..

[102] Kim S., Kwon J., Kim S., Lee B. (2001). Multiplexed strain sensor using fiber grating-tuned fiber laser with a semiconductor optical amplifier. IEEE Photonics Technol. Lett..

